# Identification and classification of known and putative antimicrobial compounds produced by a wide variety of Bacillales species

**DOI:** 10.1186/s12864-016-3224-y

**Published:** 2016-11-07

**Authors:** Xin Zhao, Oscar P. Kuipers

**Affiliations:** 1 Department of Molecular Genetics, University of Groningen, Nijenborgh 7, Groningen, 9747AG The Netherlands; 2 School of Chemical Engineering and Technology, Tianjin University, Tianjin, 300072 People’s Republic of China

**Keywords:** Antimicrobials, Bacillales, *Bacillus*, Genome-mining, Lanthipeptides, Sactipeptides, Thiopeptides, NRPs, PKs

## Abstract

**Background:**

Gram-positive bacteria of the Bacillales are important producers of antimicrobial compounds that might be utilized for medical, food or agricultural applications. Thanks to the wide availability of whole genome sequence data and the development of specific genome mining tools, novel antimicrobial compounds, either ribosomally- or non-ribosomally produced, of various Bacillales species can be predicted and classified. Here, we provide a classification scheme of known and putative antimicrobial compounds in the specific context of Bacillales species.

**Results:**

We identify and describe known and putative bacteriocins, non-ribosomally synthesized peptides (NRPs), polyketides (PKs) and other antimicrobials from 328 whole-genome sequenced strains of 57 species of Bacillales by using web based genome-mining prediction tools. We provide a classification scheme for these bacteriocins, update the findings of NRPs and PKs and investigate their characteristics and suitability for biocontrol by describing per class their genetic organization and structure. Moreover, we highlight the potential of several known and novel antimicrobials from various species of Bacillales.

**Conclusions:**

Our extended classification of antimicrobial compounds demonstrates that Bacillales provide a rich source of novel antimicrobials that can now readily be tapped experimentally, since many new gene clusters are identified.

**Electronic supplementary material:**

The online version of this article (doi:10.1186/s12864-016-3224-y) contains supplementary material, which is available to authorized users.

## Background

Most of the species of the genus *Bacillus* and related Firmicutes are Gram-positive, aerobic endospore-forming and rod-shaped bacteria, which are found in diverse environments such as soil and clay, rocks, dust, aquatic environments, on vegetation, in food and in the gastrointestinal tracts of various insects and animals [1]. Antimicrobial compounds have been used for a variety of purposes, such as delaying spoilage by plant pathogens in agriculture and extending product shelf life in the food industry [2, 3]. In particular, *Bacillus* strains are known to produce a wide variety of biocontrol metabolites, including the ribosomally synthesized antimicrobial peptides (bacteriocins) [4], as well as non-ribosomally synthesized peptides (NRPs) and polyketides (PKs) [5].

The discovery of biosynthetic gene clusters of antimicrobial compounds by genome mining is a rewarding task, because this methodology can lead to the identification and subsequent isolation of novel molecules of pharmacological and biotechnological interest [6]. Various powerful tools with broad databases have been created for the automated screening of bacteriocin gene clusters. BAGEL3 (http://bagel.molgenrug.nl/) is a versatile fast genome-mining tool valid not only for modified- and non-modified bacteriocins, but also for non-bactericidal ribosomally produced and posttranslationally modified peptides (RiPPs) [7]. The corresponding database [7] contains all the records belonging to one of the three classes of proteins being core to BAGEL3: Class I contains RiPPs of less than 10 kDa, which currently is divided into more than 12 supported subclasses; Class II contains unmodified peptides not fitting the criteria of the first database; Class III contains antimicrobial proteins larger than 10 kDa. BAGEL3 uses DNA nucleotide sequences in FASTA format as input; multiple sequence entries per file are allowed. The input DNA sequences are analyzed in parallel via two different approaches; one is the context of bacteriocin- or RiPP gene-based mining, the other is precursor (structural gene)-based mining directly by Glimmer, which increases the success rate and lowers the need for manual evaluation of results [7]. The output is visualized in an html page, by a table of putative bacteriocins or modified peptides classified into the detailed bacteriocin class found in the mining sequence; graphics of gene clusters; annotation of each ORF in the context; as well as detailed information of putative bacteriocins, such as BLAST hits in the bacteriocin database, or the pI (Isoelectric point) value. A detailed prediction of the gene clusters of NRPs, PKs and other antimicrobials is provided by antiSMASH (http://antismash.secondarymetabolites.org), a web server and stand-alone tool for the automatic genomic identification and analysis of biosynthetic gene clusters [8–10]. A database of classes specific for many types [10] of biosynthesis signature genes is constructed by Hidden Markov Models (pHMMs) covering a wide range of known or putative secondary metabolite compounds. The antiSMASH web server allows uploading of sequence files of not only a variety of types (FASTA, GBK, or EMBL files), but also GenBank/RefSeq accession numbers. Gene clusters are first predicted and identified by Glimmer and pHMMs, respectively. Subsequently, several downstream analyses can be performed by different modules: NRPS/PKS domain analysis and annotation; prediction of the core chemical structure of PKSs and NRPSs; ClusterBlast gene cluster comparative analysis; active enzyme site analysis; and secondary metabolism Clusters of Orthologous Groups (smCOG) analysis. Moreover, the ClusterFinder algorithm is used to detect putative gene clusters of unknown types. Finally, an html output is generated and putative gene clusters are listed in a Table [8–10]. Further details including gene cluster description, annotation, percentage of gene homology with known gene clusters or published genome sequences; genomic loci for this biosynthetic pathway are shown by clicking on the related words. Biochemical properties of the putative compounds are also predicted, especially chemical structures of NRPs and PKs. Results, stored in an EMBL/XLS/GenBank/BiosynML file, can be downloaded for additional analysis.

**Fig. 1:**
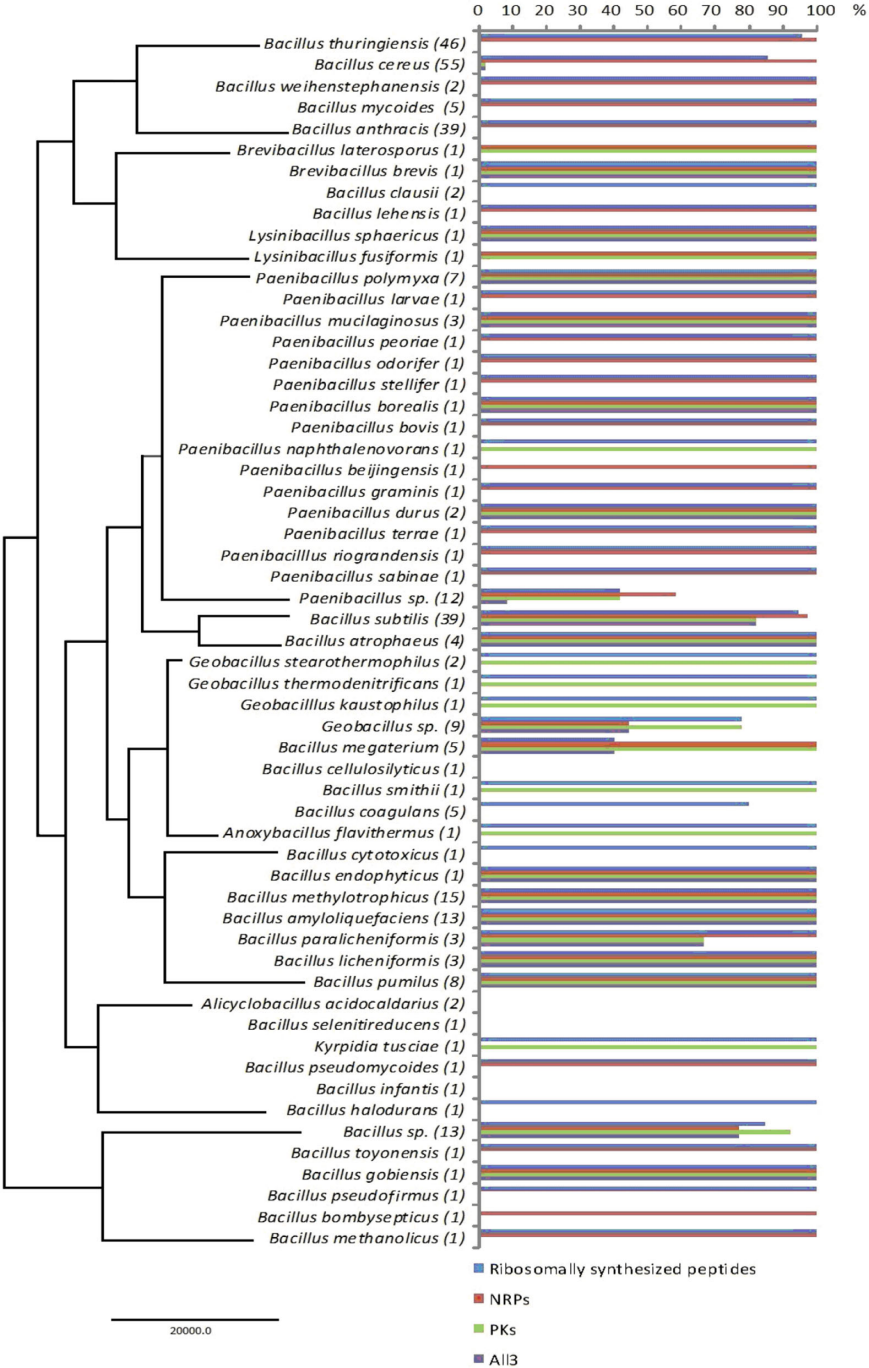
Potential of different Bacillales for ribosomally synthesized peptides, NRPs and PKs production. Phylogenetic tree was constructed by bi-directional BLAST all proteins of all genome of 328 Bacillales strains using Proteinortho; the newick tree was generated by p02tree and visualized using FigTree v1.4.3 (http://tree.bio.ed.ac.uk/software/figtree/). The percentage of strains harboring putative gene clusters of different antimicrobial compounds was calculated. Numbers in parentheses () indicate the number of genomes analyzed per genus

Although a description of *Bacillus subtilis* antimicrobials has been made before (excellent review of Stein [11]), we aim to give an updated overview and classification of bacteriocins covering various species of Bacillales, as well as NRPs and PKs, by genome mining of 57 different species within 328 whole-genome sequenced strains of Bacillales reported before March 2016 (Table 1, Additional file 1: Table S1 and Fig. 1). We also highlight examples of each class by describing the genetics and structure, with a keen eye on biocontrol properties and applications. Within the genus *Bacillus*, *B. subtilis*, *B. amyloliquefaciens*, *B. licheniformis*, *B. cereus* and *B. thuringiensis* are the best studied species for antimicrobials production [12]. Genome mining and subsequent analyses and classification of antimicrobials of other less explored Bacillales, including *Paenibacillus*, *Brevibacillus*, *Alicyclolacillus*, *Anoxybacillus*, *Lysinibacillus* and *Geobacillus* will be also included in this analysis, revealing interesting new features and distributions.

**Table 1:** Number of putative antimicrobial gene clusters identified in 328 Bacillales genomes (reported in Genbank)

Class	RPs^a^	RPs	RPs	RPs	RPs	RPs	RPs	RPs	RPs	RPs	RPs	NRPs	PKs
	I	I	I	I	I	I	I	I	II	III	TOTAL		
Genera	Lanthipeptides type I	Lanthipeptides type II	Head to tail cyclized peptides	Sacti-peptides	Glyco-cins	Lasso peptides	LAPs	Thio-peptides					
*Bacillus subtilis* (39)	6			53	15		1	1	8		84	168	66
*Bacillus thuringiensis* (46)	4	8	6	5	3	4	24		31	14	99	152	
*Bacillus anthracis* (39)							25				25	117	
*Bacillus cereus* (55)	1	16	11	4	1	9	34	3	22	4	105	144	1
*Bacillus amyloliquefaciens* (13)		16					1		12	1	30	59	48
*Bacillus licheniformis* (3)		1				3					4	6	3
*Bacillus coagulans* (5)			6							1	7		
*Bacillus megaterium* (5)	1					1					2	5	5
*Bacillus pumilus* (8)			8	2					14		24	17	8
*Bacillus atrophaeus* (4)				5			4				9	20	8
*Bacillus weihenstephanensis* (2)					1	1					2	8	
*Bacillus mycoides* (5)	2	4				5	5		4		20	14	
*Bacillus cytotoxicus* (1)							1				1		
*Bacillus clausii* (2)	1			2							3		2
*Bacillus halodurans* (1)		2								1	3		
*Bacillus cellulosilyticus* (1)													
*Bacillus infantis* (1)													
*Bacillus selenitireducens* (1)													
*Bacillus methylotrophicus* (15)		7					4		15		26	68	58
*Bacillus paralicheniformis* (3)		3	3			3					9	12	2
*Bacillus methanolicus* (1)							1				1		1
*Bacillus endophyticus* (1)		2				1	1				4	2	1
*Bacillus smithii* (1)				1							1		1
*Bacillus pseudomycoides* (1)		1				1	1		1		4	3	
*Bacillus pseudofirmus* (1)							1				1		
*Bacillus bombysepticus* (1)												4	
*Bacillus lehensis* (1)					1						1	4	
*Bacillus toyonensis* (1)						1	1		1		3	3	
*Bacillus gobiensis* (1)			1				1				2	2	1
*Bacillus* sp. (13)	3	1	3	5	1	1	2		6	2	24	36	24
*Kyrpidia tusciae* (1)			3								3		1
*Alicyclobacillus acidocaldarius* (2)													
*Anoxybacillus flavithermus* (1)									2		2		1
*Geobacillus stearothermophilus* (2)			1	1			1				3		2
*Geobacillus thermodenitrificans* (1)	1	1									2		1
*Geobacilllus kaustophilus* (1)	1		1								2		1
*Geobacillus* sp. (9)			5		1		2		1		9	3	6
*Lysinibacillus sphaericus* (1)								1			1	3	1
*Lysinibacillus fusiformis* (1)												1	1
*Brevibacillus laterosporus* (1)				1			1		1		3	5	
*Brevibacillus brevis* (1)							1		1		2	3	3
*Paenibacillus polymyxa* (7)	11	3				7					21	39	7
*Paenibacillus larvae* (1)	1		2	2					1	1	7	4	
*Paenibacillus mucilaginosus* (3)			2				3				5	10	4
*Paenibacillus peoriae* (1)	1					1					2	6	
*Paenibacillus odorifer* (1)				1							1	1	
*Paenibacillus stellifer* (1)						1					1	1	
*Paenibacillus borealis* (1)						1					1	1	2
*Paenibacillus bovis* (1)						1					1	4	
*Paenibacillus naphthalenovorans* (1)							1				1		1
*Paenibacillus beijingensis* (1)												1	
*Paenibacillus graminis* (1)				1							1	4	
*Paenibacillus durus* (2)	1	3				2			1		7	4	1
*Paenibacillus terrae* (1)						1					1	3	
*Paenibacilllus riograndensis* (1)				1							1	4	
*Paenibacillus sabinae* (1)						1					1	1	
*Paenibacillus* sp. (12)		3		3	1	3	1				11	22	6
Total	34	71	52	87	24	48	117	5	121	24	583	964	267

Numbers in parentheses () indicate the number of genomes analyzed per genus

^a^RPs is short for ribosomally synthesized peptides

## Results

### Classification of antimicrobial peptides encountered in Bacillales

The main classification scheme for ribosomally synthesized antimicrobial peptides currently available is that of the lactic acid bacteria (LAB) bacteriocins [13], which was recently reviewed and revised by Alvarez-Sieiro et al. [14]. The main classification scheme for RiPPs (Class I) was provided by the paper of Arnison et al. [15]. Although some bacteriocins produced by *Bacillus* are similar to those of LAB’s, the *Bacillus* antimicrobial compound classification system now is lagging behind that of LAB classifications. Conveniently, BAGEL3 can be used for mining bacteriocin gene clusters, some of which were not identified before. Moreover, some cryptic gene clusters of bacteriocins were identified that have not been isolated yet from wild type microorganisms. In this study, we identified 583 putative bacteriocin gene clusters from 328 strains of 57 species of Bacillales (Table 1), and these gene clusters were further classified into three classes harboring 46 types of bacteriocins covering 50 species of Bacillales (Additional file 2: Table S2) according to their gene organization and the homologies of their structural and biosynthetic genes. In addition to the published bacteriocins, many novel putative bacteriocin gene clusters were discovered. Combining this with the genome mining results of antiSMASH, we also address the non-ribosomally synthesized and polyketide synthesized antimicrobial compounds. In total 1231 putative non-ribosomal antimicrobial gene clusters were detected and subgrouped into 23 types of NRPs, five types of PKs and three types of NRPS/PKS hybrid synthesized compounds distributed over 49 species of Bacillales (Additional file 3: Table S3). In the following sections, we will describe the various classes of ribosomally synthesized peptides, NRPs, PKs and other antimicrobials present in Bacillales and indicate their presence in the various genomes.

### Ribosomally synthesized antimicrobial peptides

The classification system used in this paper for *Bacillus* ribosomally synthesized antimicrobial peptides (Table 1) comprises the major Class I: small RiPPs (based on Arnison et al.) [15] Class II: unmodified bacteriocins; Class III: large antimicrobial proteins (see also Alvarez-Sieiro et al. [14]). Characteristics of the identified bacteriocins of Bacillales are listed in Additional file 2: Table S2, describing their precursor sequences, gene clusters and predicted producer species, respectively.

#### Class I: Ribosomally produced and posttranslationally modified peptides (RiPPs)

This class consists of antimicrobial peptides (less than 10 kDa) that are ribosomally synthesized, undergoing posttranslational modifications (PTMs), resulting in different structures and properties. In this study, we found 438 putative gene clusters of class I bacteriocins, widely distributed over 49 species of Bacillales (Table 1). According to the modification differences, this class can be subdivided into seven subclasses. Subclass 1 includes peptides with modifications typical for lantibiotics (e.g. lanthionine), while subclasses 2–7 include peptides with other unique modifications [15–17].

##### Subclass 1: Lanthipeptides

Lanthipeptides are peptides containing unusual amino acids, such as dehydroalanine/dehydrobutyrine, lanthionine/methyl-lanthionine residues, introduced by different kinds of PTMs [15]. Lanthipeptides with antimicrobial activity form the so-called lantibiotics [17], which can be subdivided into four subclasses, following the classification scheme of lanthipeptides [18]. The main differences between class I, II, III and IV lanthipeptides are the PTM enzymes involved. Class I lanthipeptides are modified by two distinct enzymes that carry out the PTM process: dehydratase LanB and cyclase LanC, while class II peptides are modified by a bifunctional lanthionine-introducing enzyme, called LanM. There are also two-component lanthipeptides consisting of two peptides, which belong to class II lanthipeptide, because they are processed by a single modifying enzyme, called LanM [19–23]. For other lanthipeptides (class III and IV), the dehydration and cyclization reactions are catalyzed by multifunctional enzymes (RamC/LabKC or LanL) or they lack significant antibiotic activity, which are not further described here [24].

Subtilin is a well-investigated class I lanthipeptide produced by *B. subtilis*, the encoding gene cluster of which is also found in the genome of *Bacillus* sp. YP1. The gene encoding subtilin encodes a 56-residue peptide precursor that is processed to yield the 32-residue mature peptide, which is structurally related to the lantibiotic nisin of *Lactococus lactis* [25]. The subtilin gene cluster includes the structural gene *spaS*, encoding its prepeptide; PTM genes *spaB* and *spaC*, encoding a dehydratase and a cyclase for lanthionine formation, respectively; transporter gene *spaT* for modified precursor export and immunity genes *spaIFEG* (Additional file 2: Table S2) [26–28]. The presubtilin will be converted to mature subtilin by serine proteases secreted by *B. subtilis* [29]. Subtilin exhibits bactericidal activity against a broad spectrum of Gram-positive bacteria, based on pore formation in the cytoplasmic membrane, using cell wall precursors such as lipid II and undecaprenyl pyrophosphate, the hydrophobic carrier module for peptidoglycan monomers, as docking module and as a central constituent of the pore [30, 31]. The class II lanthipeptide mersacidin produced by several *B. amyloliquefaciens* strains [32–34], with a more globular structure comprising 20 amino acid residues, inhibits cell wall biosynthesis by binding to lipid II [35, 36]. The mersacidin gene cluster includes the structural gene *mrsA*, two modification genes (Additional file 2: Table S2), i.e. *mrsM* coding for both dehydration and cyclation and *mrsD* coding for a C-terminal S-[(Z)-2-aminovinyl]-3methyl-D-cysteine formation enzyme, and the gene *mrsT* coding for a transporter with an associated protease domain, as well as three genes, *mrsEFG*, coding for immunity and three genes, *mrsR1, R2, K1*, coding for regulation [37–39].

**Fig. 2:**
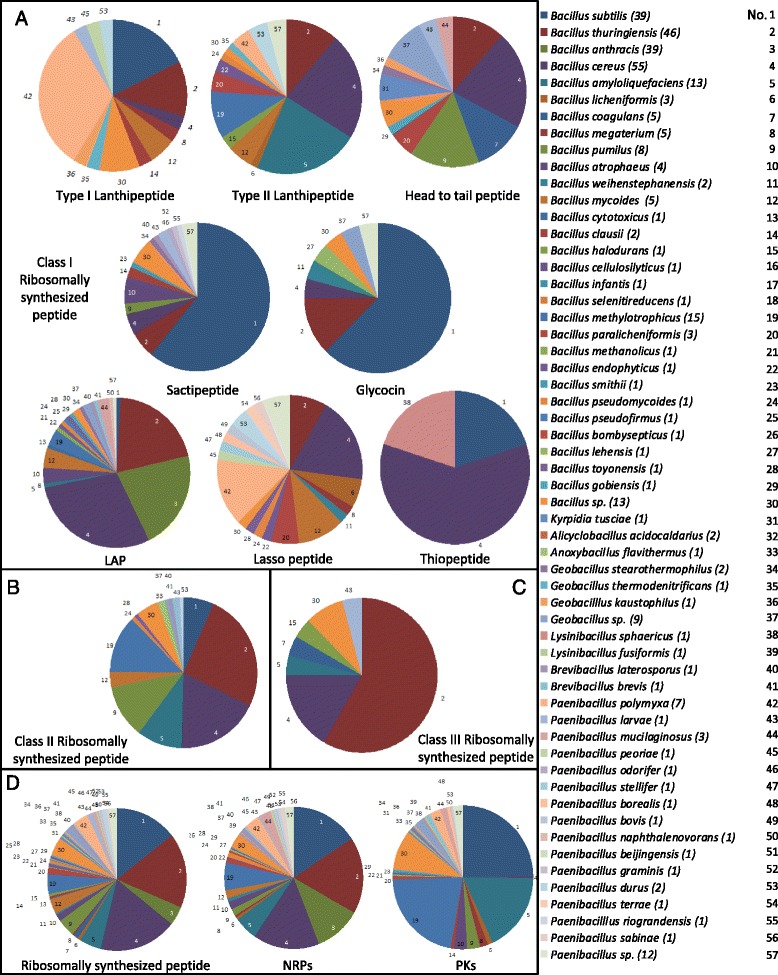
Distribution of antimicrobials biosynthetic gene clusters among different Bacillales. **a** Class I ribosomally synthesized peptides; **b** Class II ribosomally synthesized peptides; **c** Class III ribosomally synthesized peptides; **d** Total ribosomally synthesized peptides, NRPs and PKs, respectively

A total of 105 putative lanthipeptide gene clusters were discovered in Bacillales in this study (Table 1). Among them, gene clusters of class I lanthipeptides distribute over the genomes of *B. subtilis*, *B. thuringinensis*, *B. cereus*, *B. megaterium*, *B. mycoides*, *B. clausii*, *Bacillus* sp., *Geobacillus thermodenitrificans*, *Geobacillus kaustophilus*, *Paenibacillus polymyxa*, *Paenibacillus larvae*, *Paenibacillus peoriae* and *Paenibacillus durus,* while gene clusters of class II lanthipeptides distribute over the genomes of *B. thuringinensis, B. cereus*, *B. amyloliquefaciens*, *B. licheniformis*, *B. mycoides*, *B. halodurans*, *B. methylotrophicus*, *B. paralicheniformis*, *B. endophyticus*, *B. pseudomycoides*, *Bacillus* sp., *G. thermodenitrificans*, *P. polymyxa, P. durus* and *Paenibacillus* sp. (Additional file 2: Table S2 and Fig. 2). Class I lanthipeptides identified by BAGEL3 includes subtilin, clausin, subtilomycin and geobacillin I [22, 40–42]. Gene clusters of entianin, ericinA/S, paenibacillin, paenicidin A, B, thuricin 4A and its derivative thuricin 4D were not found by genome mining tools (because whole genome sequences of the producing organisms were not available in most cases) but were also added to the list (Additional file 2: Table S2) [43–47]. Class II lanthipeptides usually exhibit a globular structure, including mersacidin, amylolysin, pseudomycoicidin, cerecidin A1-A6 and geobacillin II; also two-component class II lanthipeptides including haloduracin and lichenicidin were identified [19, 22, 23, 48–52]. It is notable that gene clusters of two novel subtilin-like lantibiotics were found in several *P. polymyxa* strains. By further analysis, both of their sequence of core peptides showed high similarity with the N-terminal part of subtilin but were quite different in the C-terminal part. Moreover, we report a novel gallidermin/nisin-like lantibiotic from genomes of *Bacillus mycoides* ATCC 6462, *B. mycoides* 2048 and *B. cereus* AH1272. Looking into the sequence of its precursor peptide (see Additional file 2: Table S2), it has the conserved F(N/D)LD motif in its leader and theoretically could form the same rings as gallidermin/nisin according the position of serine and cysteine residues. All of the three putative lantibiotics have *lanBC* genes in their gene clusters, which suggest they are involved in their production. A gene cluster of a two-peptide bacteriocin was found in the genome of *B. cereus* Q1. Due to the existence of a *lanM* gene, it was predicted to be a class II lanthipeptide. Interestingly, the C-terminal parts of both its core peptides are similar to lichenicidin and haloduracin, and the N-terminal part of one of the core peptides shows high similarity with one of cytolysins produced by *Enterococcus faecalis* [53].

##### Subclass 2: Head to tail cyclized peptides

Head to tail cyclic peptides are named by their unifying feature, which is the head to tail circularization of their peptide backbones by direct linkage of their N- and C-terminal amino acids, resulting in a well-defined three-dimensional structure, by folding in α-helical manner [54–57]. To our knowledge, these peptides contain no lanthionine, β-methyl-lanthionine, and dehydrated residues, making them clearly distinguishable from lanthipeptides [58].

Amylocyclicin was recently reported to be produced by *B. amyloliquefaciens* FZB42 and identified as a novel circular bacteriocin [59], which is derived from the 112 amino acid precursor AcnA (Additional file 2: Table S2) encoded by *acnA,* with a 48 amino acid derived leader cleaved by a protease that is still unknown, and then circularization occurring between Leu-1 and Trp-64 [59]. There are gene clusters present, regulating their maturation (e.g. circularization and cleavage), transportation and self-protection. The first gene of the putative operon, *acnB,* encodes a membrane-anchored protein comprising five transmembrane helices with unkown function. *acnD* is likely to encode the transporter complex, whereas AcnC might act as circularization enzyme showing high similarity with the sequence of UclB, which brings uberlysin to maturation [60]. *Acn*EF are proposed to be the putative immunity genes. Amylocyclicin has the ability to inhibit Gram-positive bacteria like *B. subtilis*, but not against Gram-negative bacteria.

There are 52 gene clusters of putative head to tail cyclized peptides identified in this genome-mining study, which distribute over the genomes of *B. thuringiensis*, *B. cereus*, *B. coagulans*, *B. pumilus*, *B. paralicheniformis*, *B. gobiensis*, *Bacillus* sp., *Kyrpidia tusciae*, *Geobacillus stearothermophilus*, *G. kaustophilus*, *Geobacillus* sp., *P. larvae* and *Paenibacillus mucilaginosus* (Table 1 and Fig. 2). An amylocyclicin-like circular bacteriocin gene cluster was found in the genomes of *B. coagulans*. The core peptide sequence is identical to that of amylocyclicin of *B. amyloliquefaciens* FZB42, but the leader peptide sequence is quite different (Additional file 2: Table S2). It is noteworthy that a gene cluster of an uberolysin-like peptide was detected in the genome of *Bacillus* sp. 1NAL3E and gene clusters of circularin A/bacteriocin AS-48 like peptide were detected in several *Geobacillus* sp*.*, while uberolysin was produced by *Streptococcus uberis*, circularin A was produced by *Clostridium beijerinckii* and bacteriocin AS-48 was produced by *E. faecalis* [54, 60–63]. From the core peptide sequences, their circularization is most likely being formed between leucine and tryptophan (Additional file 2: Table S2). There are also other putative gene clusters of head to tail cyclized peptides found in this study, but notably these show no similarity with reported peptides. Whether these are real circular bacteriocins or not, need to be further investigated experimentally.

##### Subclass 3: Sactipeptides

Sactipeptides form a class of cyclic antimicrobial peptides with unusual sulfur to α-carbon cross-links, which are catalyzed by radical S-adenosylmethionine (SAM) enzymes in a leader peptide-dependent manner [64, 65]. Posttranslational linkage of a thiol to the α-carbon of an amino acid residue responsible for their antimicrobial bioactivities is rare in ribosomal synthesized peptides and they are classified as an independent group [66–68]. These unusual linkages differ from lanthionine bridges containing sulfur to β-carbon linkages.

Subtilosin A is a 35-residue peptide, formed by cleavage of a seven amino acid leader peptide, cyclization of the N- and C-terminal parts, and further modification of cysteine, threonine and phenylalanine residues. The maturation of subtilosin A begins with the transcription and translation of the *sbo-alb* genes (Additional file 2: Table S2), resulting in the precursor peptide SboA [69, 70]. Subsequently, the radical SAM enzyme AlbA generates the thioether linkages between the sulfur atom of the cysteine residue and the α-carbon of the threonine residue [68]. Afterwards, either AlbE or AlbF (putative proteases) cleaves off the leader peptide. In the last step, the peptide backbone is circularized by one of the two proteases, resulting in subtilosin A, which is subsequently exported by the putative ABC transporter AlbC. The operon is induced under anaerobic conditions and is controlled by the transition state regulatory protein AbrB [4]. It shows antibacterial activity against *Bacillus* spp., *E. faecalis*, *Gardnerella vaginalis* and *Listeria monocytogenes* by targeting their membranes and forming pores [71–73].

In this study, we found 87 putative gene clusters of sactipeptides in the genomes of Bacillales (Table 1), most of which belong to three reported types of sactipeptides (Additional file 2: Table S2): subtilosin A from *B. subtilis*, *B. atrophaeus*, *B. simthii* and *Bacillus* sp. strains; sporulation killing factor (SKF) from *B. atrophaeus, B. pumilus* and *B. subtilis* strains; and thuricins, such as thuricin H (17) and thuricin CD from *B. thuringiensis* and *B. cereus* [67, 74–77]. We found several other putative gene clusters of sactipeptides in the genomes of *B. clausii*, *G. stearothermophilus*, *Brevibacillus laterosporus*, *P. larvae*, *Paenibacillus odorifer*, *Paenibacillus graminis, Paenibacillus riograndensis* and *Paenibacillus* sp. (Additional file 2: Table S2 and Fig. 2), which showed very limited similarity with reported sactipeptides, and that need to be further experimentally confirmed.

##### Subclass 4: Linear azole-containing peptides (LAPs)

The linear azole-containing peptides (LAPs), form an important subgroup of RiPPs with a distinguishing heterocyclic ring of oxazoles and thiazoles derived from serine/threonine and cysteine by enzymatic cyclodehydration and dehydrogenation [78–81]. Prominent natural products such as microcin B17 produced by *Escherichia coli* and streptolysin S produced by LAB, are model of representive LAPs peptides [82–86]. The LAP family has already been extended with plantazolicin A and B produced by *B. amyloliquefaciens and B. methylotrophicus* [80, 81].

Plantazolicin A (Additional file 2: Table S2) and its desmethyl analogue plantazolicin B represent an unusual type of thioazole/oxazole-containing peptide antibiotic with a hitherto unknown mechanism of action, which show inhibition against *Bacillus* [80, 87]. The mature product plantazolicin is a linear 41 amino acid precursor peptide with the 14 amino acid core-peptide encoded by the structural gene *pznA*. The trimeric protein complex PznBCD (cyclodehydratase, dehydrogenase, and docking/scaffolding protein) likely catalyzes PTMs of ten cyclodehydrations followed by nine dehydrogenations. After the protease PznE cleaves off the leader peptide to yield desmethylplantazolicin plantazolicin B, a final N, N-bismethylation by methyltransferase PznL gives plantazolicin A [80, 81].

A total of 117 putative gene clusters of LAPs occupy 20 % of the total putative gene clusters of bacteriocins in this study and are widely distributed in more than 20 species of Bacillales (Table 1 and Fig. 2). However, only plantazolicin A and B produced by *B. amyloliquefaciens* and *B. methylotrophicus* have been reported before (Additional file 2: Table S2). This means that many novel LAPs can be found and need further experimental investigation.

##### Subclass 5: Thiopeptides

Thiopeptides, or thiazolyl peptides are highly modified via either non-ribosomal or ribosomal assembly, with a six membered nitrogenous macrocycle being central of piperidine/pyridine/dehydropiperidine and including additional thiazoles and dehydrated amino acid residues [15, 88, 89]. Because of the trithiazolyl (tetrahydro) pyridine core, they display high affinity binding to either the 50S ribosomal subunit or elongation factor Tu.

In the thiocillins, found in the producer *B. cereus* ATCC 14579, at least 10 and up to 13 of the 14 C-terminal residues undergo PTM to generate a set of eight related antibiotics. The thiocillin gene cluster contains four identical copies of a gene encoding a 52-residue precursor peptide (*tclE-H*), which is thought to be posttranslationally modified to yield the mature antibiotic scaffold (Additional file 2: Table S2). Four of the eight thiocillins produced abundantly by *B. cereus* display similar efficacies against *B. subtilis* and two methicillin-resistant *Staphylococcus aureus* (MRSA) strains [90, 91].

Thiopeptide gene clusters involved in ribosomal synthesis are found in the genome sequences of several *B. cereus*, *B. subtilis* and *Lysinibacillus sphaericus* (Additional file 2: Table S2 and Fig. 2), which might go beyond the classification for LAB bacteriocins [14].

##### Subclass 6: Glycocins

Glycocins are bacteriocins with glycosylated residues. There are various unique and diverse putative glycopeptide containing bacteriocins named glycocins in Firmicutes [15, 92].

There is one model glycopeptide bacteriocin, sublancin 168 (Additional file 2: Table S2), produced by *B. subtilis* with a β-S-linked glucose moiety attached to cysteine22 and two disulfides [92–95]. The sublancin 168 biosynthetic gene cluster contains the precursor gene *sunA* coding a 56-residue polypeptide consisting of a 19-residue leader peptide and a 37-residue mature peptide and genes *bdbA* and *bdbB* encoding two thiol-disulfide oxidoreductases, i.e. BdbA and BdbB [95, 96]. In addition, it contains two open reading frames of unknown function, *yolJ* and *yolF*. YolF was recently suggested to be important for immunity of the producing strain and was renamed SunI; the function of YolJ has not yet been reported [97]. SunT is responsible for transport. The antimicrobial activity spectrum of sublancin 168 was like that of lantibiotics, inhibiting Gram-positive bacteria, but not Gram-negative bacteria; and acts also similar to the lantibiotics nisin and subtilin in its ability to inhibit both bacterial spore outgrowth and vegetative cell growth [17].

In addition to sublancin 168 found in *B. subtilis*, genome-mining study indicated that nine other putative gene clusters of glycocins were found in genomes of *B. thuringiensis*, *B. cereus*, *B. weihenstephanensis*, *B. lehensis*, *Bacillus* sp., *Geobacillus* sp. and *Paenibacillus* sp., which need further characterization (Table 1 and Fig. 2).

##### Subclass 7: Lasso peptides

Lasso peptides, which form an emerging class of RiPPs from bacteria, were first described in 1991 [98]. Their defining structural feature is an N-terminal macrolactam ring that is threaded by the C-terminal tail resulting in a unique lasso structure–the so-called lariat knot. The ring is formed by an isopeptide bond between the N-terminal α-amino group of a glycine, alanine, serine, or cysteine and the carboxylic acid side chain of an aspartate or glutamate, which can be located at positions 7, 8, or 9 of the amino acid sequence [16, 99].

In general, lasso peptide production requires at least three genes encoding a precursor peptide A, a cysteine protease B, and an ATP-dependent lactam synthetase C. Gene clusters might contain additional genes, but so far no system was proven to be in need of an additional enzyme to produce mature lasso peptides [100–104]. Microcin J25 produced by *E. coli* AY25 has served as a model for studies of lasso peptides [105]. Known lasso peptides display antimicrobial activity by enzyme inhibition [106, 107].

Genome mining of Bacillales indicated 48 gene clusters of hypothetical peptides, which are likely lasso peptides in the genomes of 20 Bacillales species (Table 1 and Fig. 2), but these still need to be experimentally confirmed.

#### Class II: unmodified bacteriocins

Class II bacteriocins include small (less than 10 kDa), ribosomally synthesized, heat-stable, membrane-active linear peptides [4, 108, 109]. According to genome mining results, we found in total 121 putative gene clusters of class II bacteriocins distributed over 16 species of Bacillales (Table 1 and Fig. 2). This class can be subdivided into two subclasses: 1. Pediocin-like peptides; 2. Other unmodified peptides (Additional file 2: Table S2).

##### Subclass 1: Pediocin-like peptides

The pediocin-like bacteriocins are antilisterial peptides that have a YGNGVXC consensus motif [110, 111]. Coagulin produced by *B. coagulans* I_4_ is a peptide of 44 residues has an amino acid sequence similar to that described for pediocins AcH and PA-1 [109, 112]. Coagulin and pediocin differ only by a single amino acid at their C-terminus (asparagine41threonine). Gene clusters of coagulin are located on a plasmid including the structural gene *coaA*, immunity gene *coaB*, and ABC transporter genes *coaC* and *coaD* [113].

##### Subclass 2: Other unmodified peptides

Subclass 2 includes other unmodified peptides, such as lichenin produced by *B. licheniformis*, or cereins produced by *B. cereus*, which have already been described in a previous review although not yet detected in the reported complete genome sequences [4]. We found a lactobin A family protein [114] and a lactococcin A1 family protein [115] belonging to class II bacteriocins from *Anoxybacillus flavithermus* WK1. Here, we mainly added some new members of *Bacillus* class II bacteriocins detected by BAGEL3, in particular holins and holin-like peptide BhlA, antimicrobial peptide LCI, and leaderless bacteriocin aureocin A53 (Additional file 2: Table S2).

Analysis of all Bacillales genome sequences revealed the presence of a structural gene encoding a holin in *Geobacillus* sp. WCH70 and BhlA encoding genes in most of *B. subtilis*, *B. amyloliquefaciens*, *B. mycoides*, *B. pseudomycoides, B. licheniformis, B. pumilus* and *B. thuringiensis*, and further structural analysis of their sequence revealed features similar to holin (Additional file 2: Table S2) [116, 117]. Holins are phage-encoded proteins involved in the disruption of bacterial membrane to facilitate the release of progeny phage particles [118–121]. However, the functions of these specific ORFs have not yet been identified. The bacteriocin-related holin-like peptide BhlA from *Bacillus* showed antibacterial activity against several Gram-positive bacteria, including MRSA and *Micrococcus luteus* by destroying cell membranes [122]. BhlA consists of 70 amino acid residues with a single transmembrane domain at the N-terminus, a number of highly charged amino acid residues at the C-terminus. The presence of hydrophilic residues and the membrane topology of BhlA make it different from holins [122].

The *lci* gene encoding LCI was found in the genomes of *B. amyloliquefaciens* and *B. methylotrophicus* strains (Additional file 2: Table S2), sharing 98–100 % identity with the LCI sequence of *B. subtilis*. The antimicrobial peptide LCI was first identified and isolated by Liu et al. [123] from a *B. subtilis* strain named A014 that possesses very strong antagonistic activity against the Gram-negative pathogen *Xanthomonas campestris pv oryzea* causing rice leaf-blight disease, which is a serious threat to rice production and causes great losses in yields in most rice fields annually. LCI is a β-structure antimicrobial peptide containing 47 residues of 5460 Da with no disulfide bridge or circular structure. It also contains a hydrophobic core formed by valine5, tyrosine41 and tryptophan44 as well as 23 H-bonds which contribute to its considerable thermal stability [124, 125]. According to our BAGEL3 gene cluster mining results, there are two genes: a structural gene *lci* and an immunity/transporter-like gene which was still unknown. LCI’s positively charged residues lead to a short-lived channel in the bacterial membrane of sensitive strains [126].

Another new member of *Bacillus* class II bacteriocins is leaderless aureocin A53, whose gene cluster was identified in the genome sequence of *B. pumilus* strains (Additional file 2: Table S2). It is active against *L. monocytogenes* by dissipating the membrane potential and simultaneously stopped biosynthesis of DNA, polysaccharides, and protein [127]. Aureocin A53 is a highly cationic 49-residue peptide containing six lysine and four tryptophan residues. Unlike most class II bacteriocins, aureocin A53 is synthesized without a leader peptide and retains a formylated N terminus. Notably, genes for biosynthetic enzymes, immunity functions, or regulation of biosynthesis are not found in the vicinity of the aureocin A53 structural gene [128].

#### Class III: large antimicrobial proteins

This group includes large proteins (larger than 10 kDa) with antimicrobial activity. Gene clusters of these proteins normally include an immunity gene and a structural gene [126]. We found 24 putative gene clusters of class III bacteriocins distributed over seven species of Bacillales (Table 1 and Fig. 2). In a previous review, megacins produced by *B. megaterium* ATCC 19213 were reported as class III bacteriocins [4, 129]. Here, we identified and introduced some class III bacteriocins by BAGEL3 respresented by colicin, M23 peptidase and pyocin AP41 (Additional file 2: Table S2).

Gene clusters of colicins were identified in the genomes of *B. thuringiensis*, *B. cereus* and *Bacillus* sp. BH072 (Additional file 2: Table S2). Channel-forming colicins (colicins A, B, E1, Ia, Ib, and N) are transmembrane proteins that depolarize the cytoplasmic membrane, leading to dissipation of cellular energy. Their immunity gene is often produced constitutively, while the bacteriocin release protein is generally produced only as a read-through of the stop codon on the colicin structural gene. The colicin itself is repressed by the SOS response and may be regulated in other ways, as well [130]. Pyocin AP41 is also discovered as a large bacteriocin from *B. thuringiensis* (Additional file 2: Table S2), which was first isolated from *Pseudomonas aeruginosa* PAF41. According to literature, it showed a similar mode of action to that of colicin [131]. Interestingly, we found gene clusters of M23 peptidase in the genomes of *B. thuringiensis*, *B. coagulans* and *B. halodurans* (Additional file 2: Table S2), while M23 peptidase has not been reported to be secreted by *Bacillus* before, and so needs to be further experimentally confirmed. Over the past years, many members of the M23 metallopeptidase family have been identified and biochemically characterized. Structures have been determined for some of them, e.g. LytM, LasA and recently lysostaphin, a prototypic enzyme of the M23B group and the best studied bacteriocin of this group [132, 133].

### Non-ribosomal synthesized peptides (NRPs) and polyketides (PKs) of Bacillales

NRPs and PKs encompass a variety of linear, cyclic and branched structures, which are generated by complex enzymes known as non-ribosomal peptide synthetases (NRPS), polyketide synthetases (PKS) and hybrid NRPS/PKS, respectively [134, 135]. Among them, NRPs produced by Bacillales include lipopeptides (LPs) and others, with significant antimicrobial activity [136]. Here we present an extended collection based on members described in a previous review by Aleti et al. [136]. By use of antiSMASH, we identified 31 types of putative NRPs, PKs and NRPS/PKS hybrid synthesized antimicrobials, which will be described in detail below. Characteristics of them are listed in Additional file 3: Table S3 by displaying their chemical structures, gene clusters and predicted producer species, respectively.

#### Lipopeptides (LPs)

Lipopeptides (LPs) are natural compounds of bacterial origin consisting of a hydrophobic long alkyl chain linked to a hydrophilic polypeptide to form a cyclic or linear structure [137]. According to our mining results, *B. amyloliquefaciens*, *B. methylotrophicus*, *B. atrophaeus*, *B. subtilis*, *B. licheniformis*, *B. paralicheniformis*, *B. pumilus, B. lehensis, B. laterosporus, Bacillus* sp., *P. polymyxa, P. larvae*, *P. mucilaginosus*, *P. peoriae*, *P. bovis*, *Paenibacillus terrae* and *Paenibacillus* sp. are likely to be the main producers of LPs, which are mainly known for their antifungal properties [138–140]. Based on a previous genome mining work (see review Aleti et al. [136]), we identified locillomycins as novel members of LPs in species of Bacillales (Additional file 3: Table S3).

Traditional LPs (comprising the surfactins, iturins and fengycins) from *Bacillus* are homologues differing in length, branching, and saturation of their acyl chain. The surfactin family (exemplified by surfactin, lichenysins and pumilacidins) contain a cyclic heptapeptide that forms a lactone bridge with ß hydroxy fatty acids [141]. The iturin group includes A, C, D and E isoforms, bacillomycin D, F and L and mycosubtilin. All these compounds contain a cyclic heptapeptide acylated with ß amino fatty acids [142, 143]. The fengycin family comprises the decapeptide fengycin A and fengycin B, which differ in a single amino acid at the sixth position (D-alanine and D-valine, respectively) [144]. Kurstakins form another family of LPs composed of four partially cyclic heptalipopeptides, which differ only in their fatty acid chains [145]. The gene clusters of the *Bacillus* LPs encoding the surfactin, fengycin, iturin and kurstakin families have been described and summarized in a number of recent reviews [6, 11, 136, 145]. Cerexins are linear LPs with strong antimicrobial activity against *S. aureus* and *Streptococcus pneumoniae* [146]. Kurstakins and cerexins are isolated and identified from *B. thuringiensis* and *B. cereus* strains before, respectively [146, 147]. Locillomycins (locillomycin A, B, and C derivatives), a novel family of cyclic lipopeptides active against bacteria and viruses produced by *B. subtilis* 916 [148, 149], include a unique nonapeptide sequence and macrocyclization. The locillomycin biosynthetic gene cluster encodes four proteins (LocA, LocB, LocC, and LocD) that form a hexamodular NRPS to biosynthesize cyclic nonapeptides.

*Paenibacillus* now are found to produce a large number of LPs [136]. Polymyxins are cyclic cationic LPs which contain the non-proteogenic amino acid 2, 4-diaminobutyric acid contributing to the overall positive charge of the cationic LPs, exhibiting antibacterial activity against both Gram-positive and Gram-negative bacteria by acting on their membranes. The gene cluster consists of five genes, of which *pmxA*, *B* and *E* encode the polymyxin synthetase, whereas *pmxC* and *D* are involved in transport [136, 150]. Another cationic lipopeptide, paenibacterin is a new broad-spectrum antimicrobial agent consisting of a cyclic 13-residue peptide and an N-terminal C15 fatty acyl chain [151]. There are also cyclic noncationic LPs from *Paenibacillus* comprising fusaricidins containing a cyclic hexapeptide structure with antagonistic activity against *Fusarium oxysporum*, tridecaptins with strong antimicrobial activity against Gram-negative bacteria. Polypeptins, octapeptins, pelgipeptins, gavaserin and saltavalin are LPs isolated from *Paenibacillus* sp. strains, reported before by other scientists, and should also be included in this collection [136, 152–155].

#### Other NRPs

By antiSMASH, we also found non-lipopeptide but NRPSs gene clusters putatively encoding NRPs with antimicrobial activity mainly in the species of *Bacillus*, *Paenibacillus* and *Brevibacilllus*. We collected them as a group of other NRPs, which is exemplified by the following NRPs (Additional file 3: Table S3).

The non-ribosomal dodecapeptide bacitracin, released by some *B. licheniformis* and *B. subtilis* strains, proved to be an inhibitor of cell wall biosynthesis of Gram-positive bacteria [156, 157]. Small peptide bacilysin secreted by *B. subtilis*, *B. amyloliquefaciens* and *B. pumilus* contains an N-terminal alanine residue and L-anticapsin with antibacterial activity against *S. aureus* [158]. *B. subtilis* also produces rhizocticins, phosphonate oligopeptide antibiotics containing the C-terminal non-proteinogenic amino acid (Z)-1-2-amino-5-phosphono-3-pentenoic acid displaying antifungal activity [159]. Petrobactin and bacillibactin produced by several *Bacillus* strains under iron-limited conditions, are catecholate siderophores associated with two operons, *asb* (for petrobactin) and *bac* (for bacillibactin) [160].

Sevadicin is a tripeptide (D-phenylalanine-D-alanine-tryptophan) produced by a NRPS encoded by a gene cluster found in the genome of *P. larvae*, which was shown to have antibacterial activity [161].

Both the cyclic peptides gramicidin S and tyrocidine, produced by *Brevisbacillus*, consist of 10 amino acid residues [162]. Gramicidin S consists of two identical pentapeptides, which are linked head to tail, and together form the stable amphiphilic cyclic decapeptide. The first amino acid residue of the two pentapeptides is in the D-configuration [163]. The peptide exhibits strong antibacterial and antifungal activity [164]. Tyrocidine, actually a mixture of slightly different decapeptides, is active against several Gram-positive bacteria and it has been suggested that this peptide plays a role in the regulation of sporulation of *B. brevis* [162]. The gramicidin S biosynthesis operon (*grs*) contains thee genes, which are *grsA*, encoding the gramicidin S synthetase 1; *grsB*, encoding the gramicidin S synthetase 2, and *grsT*, encoding a protein of unknown function. The sequence of the *grsA* gene product showed a high similarity with the tyrocidine synthetase 1 (TycA protein) [165, 166].

#### Polyketides (PKs)

Polyketides represent a group of secondary metabolites, exhibiting remarkable diversity both in terms of their structure and function. Polyketide natural products are known to possess a wealth of pharmacologically important activities, including antimicrobial, antifungal, antiparasitic, antitumor and agrochemical properties (http://www.nii.ac.in/~pksdb/polyketide.html). Novel gene clusters likely encoding similar PKSs were identified using antiSMASH. They were most prominent in *B. subtilis*, *B. amyloliquefaciens*, *B. methylotrophicus*, *B. atrophaeus*, *B. laterosporus* and *Paenibacillus* sp. (Additional file 3: Table S3 and Fig. 2). The genus *Bacillus* produces three types of PKs including bacillaene, difficidin and macrolactin; *Paenibacillus* produces paenimacrolidin [6, 167]. *B. laterosporus* also produced the polyketide basiliskamide with antifungal activity [168], and it was added as novel member of PKs in species of Bacillales (Additional file 3: Table S3).

Bacillaene was first isolated from *B. subtilis* strains [169], are found to display a linear structure comprising a conjugated hexaene, while its gene clusters *bae* (*baeJ, L, M, N* and *R*) has now been discovered in several other *Bacillus* genomes, including *B. amyloliquefaciens*, *B. atrophaeus* and *P. polymyxa*. It is an inhibitor of prokaryotic protein synthesis, constituted by an open-chain enamine acid with an extended polyene system and shows good antimicrobial activity against human pathogens such as *Serratia marcescens*, *Klebsiella pneumoniae* and *S. aureus* [37, 169, 170]. Difficidin is known to be produced by *B. amyloliquefaciens* strains, which is active against the phytopathogen *Erwinia amylovora* causing fire blight, and contains a highly unsaturated macrocyclic polyene comprising a 22 membered carbon skeleton with a phosphate group rarely found in secondary metabolites [171]. Difficidin is encoded by the gene cluster *dif* with 14 open reading frames from *difA* to *difN* and *difY*. The contribution of the genes *difJ* and *difK* are unclear and their potential activities are not seen in the final product [172]. Macrolactin has also been isolated from *B. amyloliquefaciens* strains [173]. Most macrolactins consist of a 24 membered lactone ring with three diene moieties in the carbon backbone, which is encoded by the gene cluster *mln*, containing nine operons including *mlnA-I* [174]. As the other *Bacillus* polyketides, macrolactins show antibacterial activity and might have the potential to be used in medical application. Moreover, they could inhibit the proliferation of murine melanoma cancer cells and the replication of mammalian Herpes simplex virus and HIV in lymphoblast cells [136, 173]. Paenimacrolidin was isolated from *Paenibacillus* sp. F6-B70 with a 22 membered lactone ring showed high similarity with difficidin, which has antimicrobial activity against *Staphylococcus* [167]. The polyketide antibiotics basiliskamides A and B, which exhibit potentactivity against *Candida albicans* and *Aspergillus fumigatus*, both comprise a 21 membered carbon skeleton, structurally identical in every respect, except for the position of the cinnamate ester: C9 in basiliskamide A and C7 in basiliskamide B [175, 176].

#### NRPS/PKS hybrid synthesized compounds

There are three NRPS/PKS hybrid synthesized NRPs or PKs of Bacillales identified in this study (Additional file 3: Table S3). Paenilarvins are iturinic LPs exhibiting strong antifungal activities [177–180]. Paenilarvin A and B were first isolated from *P. larve* strain, whose NRPS gene clusters showed similarities with those of the iturin family LPs [180]. Zwittermicin A is also a hybrid polyketide-nonribosomal peptide produced by certain *B. cereus* group strains, inhibiting certain Gram-positive, Gram-negative, and eukaryotic microorganisms [181, 182]. Paenilamicin is another hybrid NRPS/PKS synthesized peptide with antibacterial and antifungal activity, whose encoded gene clusters (*pam*) were found the genomic sequence of the Gram-positive bacterium *P. larvae* [183].

In this study, 10 novel gene clusters encoding putative NRPs, PKs or NRPS/PKS hybrids were predicted from the genome of *B. brevis* NBRC 100599, *B. cereus* AH820, *B. cereus* G9842, *B. cereus* B4264, *B. cereus* E33L, *B. thuringiensis* HD771, *B. thuringiensis* HD789, *B. amyloliquefaciens* DSM7, *B. amyloliquefaciens* CC178, *B. methylotrophicus* NAU-B3, *B. anthracis* str. A0248, *B. anthracis* str. H9401 and *Bacillus* sp. BH072. The identified gene clusters (uncharacterized) show limited homology with gene clusters in the integrated databases. Related genes encoding the biosynthesis, predicted structures and antimicrobial activity of these compounds deserve to be experimentally validated.

## Discussion

An extensive investigation of 328 published whole genome sequences of Bacillales for the presence of ribosomally synthesized antimicrobials, NRPs or PKs encoding genes, revealed that most species of the genus *Bacillus*, *Paenibacillus* and *Geobacillus* have good potential to produce a wide variety of antimicrobials and there is a high occurrence of putative biosynthetic gene clusters. The ability of *Bacillus* from different species to produce putative antimicrobial compounds relate to their phylogenetic relationship. According to the phylogenetic tree (Fig. 1), Bacillales are divided into several groups. Among them, the group of *B. subtilis* and *B. atrophaeus*, the group of *B. amyloliquefaciens*, *B. methylotrophicus*, *B. paralicheniformis*, *B. licheniformis*, *B. pumilus* and *B. endophyticus* are excellent producers of all the three kinds of antimicrobials. Additionally, the *B. cereus* group, *Paenibacillus* strains are rich sources of bacteriocins and NRPs, while *Geobacillus* strains mainly produce bacteriocins and PKs.

More than 89 % strains covering 50 species have a predisposition towards producing ribosomally synthesized peptides (Fig. 1), some gene clusters of which show similarity with those of known bacteriocins, while some are uncharacterized or show limited homology. When it comes to the distribution of biosynthetic gene clusters of ribosomally synthesized antimicrobials among different Bacillales, lanthipeptides, head to tail cyclized peptides, sactipeptides, lasso peptides and LAPs of Class I are the most common types (Fig. 2), whilst glycocin and thiopeptide genes are present predominantly in *B. subtilis* and *B. cereus* strains, respectively. Gene clusters of class II and III appear to be also regularly contained within genus *Bacillus* genomes.

Although the emphasis here is on ribosomally synthesized peptide classes, several new NRPs and PKs with potential antimicrobial activity were also identified. Bacillales are potential NRPs producers, and the gene clusters are widely spread in 40 species of the sequenced genomes in our analysis (Fig. 1). In contrast, only half of the genomes of these organisms appear to have PKs encoding genes. *Bacillus* and *Paenibacillus* genera in particular are well noted for their capability to produce structurally diverse NRPs and PKs. Approximately 35 % strains of the Bacillale species analyzed have the ability to produce all three types of antimicrobial compounds simultaneously. In this study, most of the genomes (255 of 328) were completely sequenced yielding one or only a few contigs, while there are some other level sequence data (shown in Additional file 1: Table S1) composed of relatively many single contigs. Some of these contigs are not in the correct order, which can result in higher mining counts of NRPs (caused by duplications or multiplications) than actual correct. In order to avoid this overestimation, the numbers of putative gene clusters of NRPs identified in Bacillales genomes, especially for *B. cereus* group strains, were adjusted by removing duplications or multiplications of NRPs manually. It is valuable to take this issue into account in further and future data mining and analyses.

The massive numbers of bacteria with whole genome sequence data and the development of various specific genome mining tools have made it possible to identify an informative set of putative antimicrobial gene clusters across the genomes that can be developed into new antimicrobials. Novel information found in this genome mining study includes three types that are novel: class I bacteriocins with either a new leader sequence or new core sequence; known antimicrobial compounds previously produced by other microorganisms; and completely novel gene clusters that need experimental confirmation. Another value of this study is that the post-genome mining analysis includes a number of potential species never considered to be antimicrobial producers before and provide a reference for future Bacilli to be sequenced.

## Conclusions

A multitude of antimicrobial compounds have been found to be produced by a variety of *Bacillus* strains. In the past, these compounds had to be identified by intensive screening for antimicrobial activity against appropriate targets and subsequently purified using fastidious methods prior to assess their potential utilization as antibacterial or antifungal compound. Nowadays, gene clusters encoding for ribosomally produced bacteriocins, NRPs and PKs can readily be identified in the genomic sequences by genome-mining tools that not only add missing ones, but also predict novel ones. Notably, genomic tools like BAGEL3 and antiSMASH combined with specific BLAST searches, makes the identification of new compounds much easier. Although several novel gene clusters of putative antimicrobials were found, they are as yet uncharacterized and their functions remain to be studied. Our extended classification of antimicrobial compounds demonstrates that Bacillales provides a rich source of novel antimicrobials that can now be readily tapped experimentally, since many new gene clusters were identified.

## Methods

### Genome sequences

Whole genome sequences of 328 strains of Bacillales (Additional file 1: Table S1) were obtained from NCBI Genome database (http://www.ncbi.nlm.nih.gov/genome). All proteins of all genomes were compared by bi-directional BLAST using Proteinortho and newick tree file was generated by p02tree [184]. The newick tree file was visualized using FigTree v1.4.3 (http://tree.bio.ed.ac.uk/software/figtree/).

### Genome mining for gene clusters of putative antimicrobials by BAGEL 3 and antiSMASH

Genomes were analyzed for gene clusters of putative bacteriocins, NRPs, PKs or other antimicrobials by using web-based genome mining tools BAGEL3 (http://bagel.molgenrug.nl/) [7] and antiSMASH (http://antismash.secondarymetabolites.org) [8–10]. Genome mining data were collected and putative gene clusters were classified manually. By BLAST, known and novel antimicrobials were predicted and identified.

## Additional files

Additional file 1: Table S1. Accession numbers of whole genome sequences (reported in Genbank) of Bacillales analyzed in this study. (XLSX 23 kb) (XLSX 23 kb)
Additional file 2: Table S2. Characteristics of ribosomally synthesized antimicrobial peptides of Bacillales. (DOCX 201 kb)
Additional file 3: Table S3. Characteristics of NRPs, PKs and NRPS/PKS hybrid synthesized antimicrobials of Bacillales. (DOCX 854 kb)

## Abbreviations

LAB: Lactic acid bacteria
LAP(s): Linear azole-containing peptide(s)
LPs: Lipopeptides
MRSA: Methicillin-resistant *Staphylococcus aureus*
NRPs: Non-ribosomally synthesized peptides
NRPS: Non-ribosomal peptide synthetases
PKs: Polyketides
PKS: Polyketide synthetases
PTM(s): Posttranslational modification(s)
RiPPs: Ribosomally produced and posttranslationally modified peptides
SAM: S-adenosylmethionine
SKF: Sporulation killing factor

## References

[1] Nicholson WL (2002). Roles of *Bacillus* endopores in the environment. Cell Mol Life Sci.

[2] Lucera A, Costa C, Conte A, Del Nobile MA (2012). Food applications of natural antimicrobial compounds. Front Microbiol.

[3] Sumi CD, Yang BW, Yeo I-C, Hahm YT (2015). Antimicrobial peptides of the genus *Bacillus*: a new era for antibiotics. Can J Microbiol.

[4] Abriouel H, Franz CM, Ben Omar N, Galvez A (2011). Diversity and applications of *Bacillus* bacteriocins. FEMS Microbiol Rev.

[5] Finking R, Marahiel MA (2004). Biosynthesis of nonribosomal peptides1. Annu Rev Microbiol.

[6] Fickers P (2012). Antibiotic Compounds from *Bacillus*: Why are they so Amazing?. Am J Biochem Biotechnol.

[7] van Heel AJ, de Jong A, Montalban-Lopez M, Kok J, Kuipers OP (2013). BAGEL3: Automated identification of genes encoding bacteriocins and (non-)bactericidal posttranslationally modified peptides. Nucleic Acids Res.

[8] Medema MH, Blin K, Cimermancic P, de Jager V, Zakrzewski P, Fischbach MA (2011). antiSMASH: rapid identification, annotation and analysis of secondary metabolite biosynthesis gene clusters in bacterial and fungal genome sequences. Nucleic Acids Res.

[9] Blin K, Medema MH, Kazempour D, Fischbach MA, Breitling R, Takano E (2013). antiSMASH 2.0—a versatile platform for genome mining of secondary metabolite producers. Nucleic Acids Res.

[10] Weber T, Blin K, Duddela S, Krug D, Kim HU, Bruccoleri R (2015). antiSMASH 3.0–a comprehensive resource for the genome mining of biosynthetic gene clusters. Nucleic Acids Res.

[11] Stein T (2005). *Bacillus subtilis* antibiotics: structures, syntheses and specific functions. Mol Microbiol.

[12] Mondol MA, Shin HJ, Islam MT (2013). Diversity of secondary metabolites from marine *Bacillus* species: chemistry and biological activity. Mar Drugs.

[13] Klaenhammer TR (1993). Genetics of bacteriocins produced by lactic acid bacteria. FEMS Microbiol Rev.

[14] Alvarez-Sieiro P, Montalban-Lopez M, Mu D, Kuipers OP (2016). Bacteriocins of lactic acid bacteria: extending the family. Appl Microbiol Biotechnol.

[15] Arnison PG, Bibb MJ, Bierbaum G, Bowers AA, Bugni TS, Bulaj G (2013). Ribosomally synthesized and post-translationally modified peptide natural products: overview and recommendations for a universal nomenclature. Nat Prod Rep.

[16] Velasquez JE, van der Donk WA (2011). Genome mining for ribosomally synthesized natural products. Curr Opin Chem Biol.

[17] McAuliffe O, Ross RP, Hill C (2001). Lantibiotics: structure, biosynthesis and mode of action. FEMS Microbiol Rev.

[18] Knerr PJ, van der Donk WA (2012). Discovery, biosynthesis, and engineering of lantipeptides. Annu Rev Biochem.

[19] McClerren AL, Cooper LE, Quan C, Thomas PM, Kelleher NL, van der Donk WA (2006). Discovery and in vitro biosynthesis of haloduracin, a two-component lantibiotic. Proc Natl Acad Sci U S A.

[20] Takami H, Nakasone K, Takaki Y, Maeno G, Sasaki R, Masui N (2000). Complete genome sequence of the alkaliphilic bacterium *Bacillus halodurans* and genomic sequence comparison with *Bacillus subtilis*. Nucleic Acids Res.

[21] Begley M, Cotter PD, Hill C, Ross RP (2009). Identification of a novel two-peptide lantibiotic, lichenicidin, following rational genome mining for LanM proteins. Appl Environ Microbiol.

[22] Garg N, Tang W, Goto Y, Nair SK, van der Donk WA (2011). Lantibiotics from *Geobacillus thermodenitrificans*. Proc Natl Acad Sci U S A.

[23] Caetano T, Barbosa J, Moesker E, Sussmuth RD, Mendo S (2014). Bioengineering of lanthipeptides in *Escherichia coli*: assessing the specificity of lichenicidin and haloduracin biosynthetic machinery. Res Microbiol.

[24] Khusainov R, van Heel AJ, Lubelski J, Moll GN, Kuipers OP (2015). Identification of essential amino acid residues in the nisin dehydratase NisB. Front Microbiol.

[25] Lee H, Kim HY (2011). Lantibiotics, class I bacteriocins from the genus *Bacillus*. J Microbiol Biotechnol.

[26] Stein T, Heinzmann S, Kiesau P, Himmel B, Entian KD (2003). The spa-box for transcriptional activation of subtilin biosynthesis and immunity in *Bacillus subtilis*. Mol Microbiol.

[27] Kleerebezem M (2004). Quorum sensing control of lantibiotic production; nisin and subtilin autoregulate their own biosynthesis. Peptides.

[28] Kleerebezem M, Bongers R, Rutten G, de Vos WM, Kuipers OP (2004). Autoregulation of subtilin biosynthesis in *Bacillus subtilis*: the role of the spa-box in subtilin-responsive promoters. Peptides.

[29] Corvey C, Stein T, Düsterhus S, Karas M, Entian KD (2003). Activation of subtilin precursors by *Bacillus subtilis* extracellular serine proteases subtilisin (AprE), WprA, and Vpr. Biochem Biophys Res Commun.

[30] Breukink E, Wiedemann I, van Kraaij C, Kuipers OP, Sahl HG, de Kruijff B (1999). Use of the cell wall precursor lipid II by a pore-forming peptide antibiotic. Science.

[31] Parisot J, Carey S, Breukink E, Chan WC, Narbad A, Bonev B (2008). Molecular mechanism of target recognition by subtilin, a class I lanthionine antibiotic. Antimicrob Agents Chemother.

[32] Bierbaum G, Brötz H, Koller KP, Sahl HG (1995). Cloning, sequencing and production of the lantibiotic mersacidin. FEMS Microbiol Lett.

[33] Hao K, He P, Blom J, Rueckert C, Mao Z, Wu Y (2012). The genome of plant growth-promoting *Bacillus amyloliquefaciens* subsp. *plantarum* strain YAU B9601-Y2 contains a gene cluster for mersacidin synthesis. J Bacteriol.

[34] Zhao X, de Jong A, Zhou Z, Kuipers OP. Complete genome sequence of *Bacillus amyloliquefaciens* strain BH072, isolated from honey. Genome Announc. 2015;3(2):e0098–15. 10.1128/genomeA.00098-15 PMC4357757 25767235

[35] Brötz H, Bierbaum G, Reynolds PE, Sahl HG (1997). The lantibiotic mersacidin inhibits peptidoglycan biosynthesis at the level of transglycosylation. Eur J Biochem.

[36] Hsu ST, Breukink E, Bierbaum G, Sahl HG, de Kruijff B, Kaptein R (2003). NMR study of mersacidin and lipid II interaction in dodecylphosphocholine micelles. Conformational changes are a key to antimicrobial activity. J Biol Chem.

[37] He P, Hao K, Blom J, Ruckert C, Vater J, Mao Z (2012). Genome sequence of the plant growth promoting strain *Bacillus amyloliquefaciens* subsp. *plantarum* B9601-Y2 and expression of mersacidin and other secondary metabolites. J Biotechnol.

[38] Schmitz S, Hoffmann A, Szekat C, Rudd B, Bierbaum G (2006). The lantibiotic mersacidin is an autoinducing peptide. Appl Environ Microbiol.

[39] Guder A, Schmitter T, Wiedemann I, Sahl HG, Bierbaum G (2002). Role of the single regulator MrsR1 and the two-component system MrsR2/K2 in the regulation of mersacidin production and immunity. Appl Environ Microbiol.

[40] Klein C, Kaletta C, Schnell N, Entian KD. Analysis of genes involved in biosynthesis of the lantibiotic subtilin. Appl Environ Microbiol. 1992. doi: 10.1111/j.1432-1033.1992.tb16605.x. 10.1128/aem.58.1.132-142.1992 PMC195183 1539969

[41] Bouhss A, Al-Dabbagh B, Vincent M, Odaert B, Aumont-Nicaise M, Bressolier P (2009). Specific interactions of clausin, a new lantibiotic, with lipid precursors of the bacterial cell wall. Biophys J.

[42] Phelan RW, Barret M, Cotter PD, O’Connor PM, Chen R, Morrissey JP (2013). Subtilomycin: a new lantibiotic from *Bacillus subtilis* strain MMA7 isolated from the marine sponge Haliclona simulans. Mar Drugs.

[43] He Z, Yuan C, Zhang L, Yousef AE (2008). N-terminal acetylation in paenibacillin, a novel lantibiotic. FEBS Lett.

[44] van Belkum MJ, Lohans CT, Vederas JC. Draft Genome sequences of *Paenibacillus polymyxa* NRRL B-30509 and *Paenibacillus terrae* NRRL B-30644, strains from a poultry environment that produce tridecaptin A and paenicidins. Genome Announc. 2015;3(2):e00372–15. 10.1128/genomeA.00372-15 PMC4408349 25908148

[45] Fuchs SW, Jaskolla TW, Bochmann S, Kotter P, Wichelhaus T, Karas M (2011). Entianin, a novel subtilin-like lantibiotic from *Bacillus subtilis* subsp. *spizizenii* DSM 15029T with high antimicrobial activity. Appl Environ Microbiol.

[46] Stein T, Borchert S, Conrad B, Feesche J, Hofemeister B, Hofemeister J (2002). Two different lantibiotic-like peptides originate from the ericin gene cluster of *Bacillus subtilis* A1/3. J Bacteriol.

[47] Xin B, Zheng J, Xu Z, Song X, Ruan L, Peng D (2015). The *Bacillus cereus* group is an excellent reservoir of novel lanthipeptides. Appl Envrion Microbiol.

[48] Altena K, Guder A, Cramer C, Bierbaum G (2000). Biosynthesis of the lantibiotic mersacidin: organization of a type B lantibiotic gene cluster. Appl Envrion Microbiol.

[49] Herzner AM, Dischinger J, Szekat C, Josten M, Schmitz S, Yakéléba A (2011). Expression of the lantibiotic mersacidin in *Bacillus amyloliquefaciens* FZB42. Plos One.

[50] Arguelles Arias A, Ongena M, Devreese B, Terrak M, Joris B, Fickers P (2013). Characterization of amylolysin, a novel lantibiotic from *Bacillus amyloliquefaciens* GA1. Plos One.

[51] Basi-Chipalu S, Dischinger J, Josten M, Szekat C, Zweynert A, Sahl HG (2015). Pseudomycoicidin, a class II lantibiotic from *Bacillus pseudomycoides*. Appl Envrion Microbiol.

[52] Wang J, Zhang L, Teng K, Sun S, Sun Z, Zhong J (2014). Cerecidins, novel lantibiotics from *Bacillus cereus* with potent antimicrobial activity. Appl Envrion Microbiol.

[53] Coburn PS, Gilmore MS (2003). The *Enterococcus faecalis* cytolysin: a novel toxin active against eukaryotic and prokaryotic cells. Cell Microbiol.

[54] Montalbán-López M, Sánchez-Hidalgo M, Cebrián R, Maqueda M (2012). Discovering the bacterial circular proteins: bacteriocins, cyanobactins, and pilins. J Biol Chem.

[55] Maqueda M, Sanchez-Hidalgo M, Fernandez M, Montalban-Lopez M, Valdivia E, Martinez-Bueno M (2008). Genetic features of circular bacteriocins produced by Gram-positive bacteria. FEMS Microbiol Rev.

[56] Conlan BF, Gillon AD, Craik DJ, Anderson MA (2010). Circular proteins and mechanisms of cyclization. Biopolymers.

[57] Van Belkum MJ, Martin-Visscher LA, Vederas JC (2011). Structure and genetics of circular bacteriocins. Trends Microbiol.

[58] Gonzalez C, Langdon GM, Bruix M, Galvez A, Valdivia E, Maqueda M (2000). Bacteriocin AS-48, a microbial cyclic polypeptide structurally and functionally related to mammalian NK-lysin. Proc Natl Acad Sci U S A.

[59] Scholz R, Vater J, Budiharjo A, Wang Z, He Y, Dietel K (2014). Amylocyclicin, a novel circular bacteriocin produced by *Bacillus amyloliquefaciens* FZB42. J Bacteriol.

[60] Wirawan RE, Swanson KM, Kleffmann T, Jack RW, Tagg JR (2007). Uberolysin: a novel cyclic bacteriocin produced by *Streptococcus uberis*. Microbiology.

[61] Grande Burgos MJ, Pulido RP, Del Carmen Lopez Aguayo M, Galvez A, Lucas R (2014). The cyclic antibacterial peptide enterocin AS-48: isolation, mode of action, and possible food applications. Int J Mol Sci.

[62] Kawai Y, Kemperman R, Kok J, Saito T (2004). The circular bacteriocins gassericin A and circularin A. Curr Protein Pept Sci.

[63] Borrero J, Brede DA, Skaugen M, Diep DB, Herranz C, Nes IF (2011). Characterization of garvicin ML, a novel circular bacteriocin produced by *Lactococcus garvieae* DCC43, isolated from mallard ducks (Anas platyrhynchos). Appl Envrion Microbiol.

[64] Azevedo AC, Bento CB, Ruiz JC, Queiroz MV, Mantovani HC (2015). Distribution and genetic diversity of bacteriocin gene clusters in rumen microbial genomes. Appl Envrion Microbiol.

[65] Yang X, van der Donk WA (2013). Ribosomally synthesized and post-translationally modified peptide natural products: new insights into the role of leader and core peptides during biosynthesis. Chemistry.

[66] Fluhe L, Marahiel MA (2013). Radical S-adenosylmethionine enzyme catalyzed thioether bond formation in sactipeptide biosynthesis. Curr Opin Chem Biol.

[67] Kawulka K, Sprules T, McKay RT, Mercier P, Diaper CM, Zuber P (2003). Structure of subtilosin A, an antimicrobial peptide from *Bacillus subtilis* with unusual posttranslational modifications linking cysteine sulfurs to alpha-carbons of phenylalanine and threonine. J Am Chem Soc.

[68] Flühe L, Knappe TA, Gattner MJ, Schäfer A, Burghaus O, Linne U (2012). The radical SAM enzyme AlbA catalyzes thioether bond formation in subtilosin A. Nat Chem Biol.

[69] Zheng G, Yan LZ, Vederas JC, Zuber P (1999). Genes of the *sbo-alb* locus of *Bacillus subtilis* are required for production of the antilisterial bacteriocin subtilosin. J Bacteriol.

[70] Zheng G, Hehn R, Zuber P (2000). Mutational analysis of the *sbo-alb* locus of *Bacillus subtilis*: identification of genes required for subtilosin production and immunity. J Bacteriol.

[71] Noll KS, Sinko PJ, Chikindas ML (2011). Elucidation of the molecular mechanisms of action of the natural antimicrobial peptide subtilosin against the bacterial vaginosis-associated pathogen *Gardnerella vaginalis*. Probiotics Antimicrob.

[72] Sutyak KE, Wirawan RE, Aroutcheva AA, Chikindas ML (2008). Isolation of the *Bacillus subtilis* antimicrobial peptide subtilosin from the dairy product-derived *Bacillus amyloliquefaciens*. J Appl Microbiol.

[73] Huang T, Geng H, Miyyapuram VR, Sit CS, Vederas JC, Nakano MM (2009). Isolation of a variant of subtilosin A with hemolytic activity. J Bacteriol.

[74] Allenby NE, Watts CA, Homuth G, Pragai Z, Wipat A, Ward AC (2006). Phosphate starvation induces the sporulation killing factor of *Bacillus subtilis*. J Bacteriol.

[75] Lee H, Churey JJ, Worobo RW (2009). Biosynthesis and transcriptional analysis of thurincin H, a tandem repeated bacteriocin genetic locus, produced by *Bacillus thuringiensis* SF361. FEMS Microbiol Lett.

[76] Rea MC, Sit CS, Clayton E, O’Connor PM, Whittal RM, Zheng J (2010). Thuricin CD, a posttranslationally modified bacteriocin with a narrow spectrum of activity against *Clostridium difficile*. Proc Natl Acad Sci U S A.

[77] Favret ME, Yousten AA (1989). Thuricin: the bacteriocin produced by *Bacillus thuringiensis*. J Invertebr Pathol.

[78] Li YM, Milne JC, Madison LL, Kolter R, Walsh CT (1996). From peptide precursors to oxazole and thiazole-containing peptide antibiotics: microcin B17 synthase. Science.

[79] Melby JO, Nard NJ, Mitchell DA (2011). Thiazole/oxazole-modified microcins: complex natural products from ribosomal templates. Curr Opin Chem Biol.

[80] Banala S, Ensle P, Sussmuth RD (2013). Total synthesis of the ribosomally synthesized linear azole-containing peptide plantazolicin A from *Bacillus amyloliquefaciens*. Angew Chem Int Ed.

[81] Scholz R, Molohon KJ, Nachtigall J, Vater J, Markley AL, Sussmuth RD (2011). Plantazolicin, a novel microcin B17/streptolysin S-like natural product from *Bacillus amyloliquefaciens* FZB42. J Bacteriol.

[82] Davagnino J, Herrero M, Furlong D, Moreno F, Kolter R (1986). The DNA replication inhibitor microcin B17 is a forty-three-amino-acid protein containing sixty percent glycine. Proteins.

[83] Heddle JG, Blance SJ, Zamble DB, Hollfelder F, Miller DA, Wentzell LM (2001). The antibiotic microcin B17 is a DNA gyrase poison: characterisation of the mode of inhibition. J Mol Biol.

[84] Cox CL, Doroghazi JR, Mitchell DA (2015). The genomic landscape of ribosomal peptides containing thiazole and oxazole heterocycles. BMC Genomics.

[85] Lee SW, Mitchell DA, Markley AL, Hensler ME, Gonzalez D, Wohlrab A (2008). Discovery of a widely distributed toxin biosynthetic gene cluster. Proc Natl Acad Sci U S A.

[86] Nizet V, Beall B, Bast DJ, Datta V, Kilburn L, Low DE (2000). Genetic locus for streptolysin S production by group A *Streptococcus*. Infect Immun.

[87] Liu Z, Budiharjo A, Wang P, Shi H, Fang J, Borriss R (2013). The highly modified microcin peptide plantazolicin is associated with nematicidal activity of *Bacillus amyloliquefaciens* FZB42. Appl Microbiol Biotechnol.

[88] Just-Baringo X, Albericio F, Alvarez M (2014). Thiopeptide antibiotics: retrospective and recent advances. Mar Drugs.

[89] Bowers AA, Walsh CT, Acker MG (2010). Genetic interception and structural characterization of thiopeptide cyclization precursors from *Bacillus cereus*. J Am Chem Soc.

[90] Wieland Brown LC, Acker MG, Clardy J, Walsh CT, Fischbach MA (2009). Thirteen posttranslational modifications convert a 14-residue peptide into the antibiotic thiocillin. Proc Natl Acad Sci U S A.

[91] Shoji J, Hinoo H, Wakisaka Y, Koizumi K, Mayama M (1976). Isolation of three new antibiotics, thiocillins I, II and III, related to micrococcin P. Studies on antibiotics from the genus *Bacillus*. VIII. J Antibiot (Tokyo).

[92] Stepper J, Shastri S, Loo TS, Preston JC, Novak P, Man P (2011). Cysteine S-glycosylation, a new post-translational modification found in glycopeptide bacteriocins. FEBS Lett.

[93] Hsieh YS, Wilkinson BL, O’Connell MR, Mackay JP, Matthews JM, Payne RJ (2012). Synthesis of the bacteriocin glycopeptide sublancin 168 and S-glycosylated variants. Org Lett.

[94] Oman TJ, Boettcher JM, Wang H, Okalibe XN, van der Donk WA (2011). Sublancin is not a lantibiotic but an S-linked glycopeptide. Nat Chem Biol.

[95] Paik SH, Chakicherla A, Hansen JN (1998). Identification and characterization of the structural and transporter genes for, and the chemical and biological properties of, sublancin 168, a novel lantibiotic produced by *Bacillus subtilis* 168. J Biol Chem.

[96] Bolhuis A, Venema G, Quax WJ, Bron S, van Dijl JM (1999). Functional analysis of paralogous thiol-disulfide oxidoreductases in *Bacillus subtilis*. J Biol Chem.

[97] Serizawa M, Kodama K, Yamamoto H, Kobayashi K, Ogasawara N, Sekiguchi J (2005). Functional analysis of the YvrGHb two-component system of *Bacillus subtilis*: identification of the regulated genes by DNA microarray and northern blot analyses. Biosci Biotechnol Biochem.

[98] Weber W, Fischli W, Hochuli E, Kupfer E, Weibel EK (1991). Anantin—a peptide antagonist of the atrial natriuretic factor (ANF). I. Producing organism, fermentation, isolation and biological activity. J Antibiot (Tokyo).

[99] Hegemann JD, Zimmermann M, Xie X, Marahiel MA (2015). Lasso peptides: an intriguing class of bacterial natural products. Acc Chem Res.

[100] Maksimov MO, Pelczer I, Link AJ (2012). Precursor-centric genome-mining approach for lasso peptide discovery. Proc Natl Acad Sci U S A.

[101] Maksimov MO, Pan SJ, James Link A (2012). Lasso peptides: structure, function, biosynthesis, and engineering. Nat Prod Rep.

[102] Maksimov MO, Link AJ (2013). Discovery and characterization of an isopeptidase that linearizes lasso peptides. J Am Chem Soc.

[103] Solbiati JO, Ciaccio M, Farías RN, González-Pastor JE, Moreno F, Salomón RA (1999). Sequence analysis of the four plasmid genes required to produce the circular peptide antibiotic microcin J25. J Bacteriol.

[104] Yan KP, Li Y, Zirah S, Goulard C, Knappe TA, Marahiel MA, Rebuffat S (2012). Dissecting the maturation steps of the lasso peptide microcin J25 in vitro. Chembiochem.

[105] Mukhopadhyay J, Sineva E, Knight J, Levy RM, Ebright RH (2004). Antibacterial peptide microcin J25 inhibits transcription by binding within and obstructing the RNA polymerase secondary channel. Mol Cell.

[106] Helynck G, Dubertret C, Mayaux JF, Leboul J (1993). Isolation of RP 71955, a new anti-HIV-1 peptide secondary metabolite. J Antibiot (Tokyo).

[107] Delgado MA, Rintoul MR, Farias RN, Salomon RA (2001). *Escherichia coli* RNA polymerase is the target of the cyclopeptide antibiotic microcin J25. J Bacteriol.

[108] Cotter PD, Ross RP, Hill C (2013). Bacteriocins–a viable alternative to antibiotics?. Nat Rev Microbiol.

[109] Le Marrec C, Hyronimus B, Bressollier P, Verneuil B, Urdaci MC (2000). Biochemical and genetic characterization of coagulin, a new antilisterial bacteriocin in the pediocin family of bacteriocins, produced by *Bacillus coagulans* I(4). Appl Environ Microbiol.

[110] Kjos M, Borrero J, Opsata M, Birri DJ, Holo H, Cintas LM (2011). Target recognition, resistance, immunity and genome mining of class II bacteriocins from Gram-positive bacteria. Microbiology.

[111] Cui Y, Zhang C, Wang Y, Shi J, Zhang L, Ding Z (2012). Class IIa bacteriocins: diversity and new developments. Int J Mol Sci.

[112] Miller KW, Ray P, Steinmetz T, Hanekamp T, Ray B (2005). Gene organization and sequences of pediocin AcH/PA-1 production operons in *Pediococcus* and *Lactobacillus* plasmids. Lett Appl Microbiol.

[113] Hyronimus B, Le Marrec C, Urdaci MC (1998). Coagulin, a bacteriocin-like inhibitory substance produced by *Bacillus coagulans* I4. J Appl Microbiol.

[114] De Vuyst L, Avonts L, Neysens P, Hoste B, Vancanneyt M, Swings J (2004). The lactobin A and amylovorin L471 encoding genes are identical, and their distribution seems to be restricted to the species *Lactobacillus amylovorus* that is of interest for cereal fermentations. Int J Food Microbiol.

[115] Requena T, Yu W, Stoddard GW, McKay LL (1995). Lactococcin A overexpression in a *Lactococcus lactis* subsp. *lactis* transformant containing a Tn5 insertion in the *lcnD* gene. Appl Microbiol Biotechnol.

[116] Kyogoku K, Sekiguchi J (1996). Cloning and sequencing of a new holin-encoding gene of *Bacillus licheniformis*. Gene.

[117] Oki M, Kakikawa M, Nakamura S, Yamamura ET, Watanabe K, Sasamoto M (1997). Functional and structural features of the holin HOL protein of the *Lactobacillus plantarum* phage φg1e: analysis in *Escherichia coli* system. Gene.

[118] Ziedaite G, Daugelavicius R, Bamford JK, Bamford DH (2005). The Holin protein of bacteriophage PRD1 forms a pore for small-molecule and endolysin translocation. J Bacteriol.

[119] Anthony T, Chellappa GS, Rajesh T, Gunasekaran P (2010). Functional analysis of a putative holin-like peptide-coding gene in the genome of *Bacillus licheniformis* AnBa9. Arch Microbiol.

[120] Young R, Bläsi U (1995). Holins: form and function in bacteriophage lysis. FEMS Microbiol Rev.

[121] Young R (1992). Bacteriophage lysis: mechanism and regulation. Microbiol Rev.

[122] Aunpad R, Panbangred W (2012). Evidence for two putative holin-like peptides encoding genes of *Bacillus pumilus* strain WAPB4. Curr Microbiol.

[123] Liu J, Pan N, Chen Z (1990). Characterization of an anti-rice bacterial blight polypeptide LCI. Rice Genet Newsl.

[124] Gong W, Wang J, Chen Z, Xia B, Lu G (2011). Solution structure of LCI, a novel antimicrobial peptide from *Bacillus subtilis*. Biochemistry.

[125] Liu J, Li Z, Pan N, Chen Z (1992). Purification and partial characterization of an antibacterial protein LCIII. Chin J Biotechnol.

[126] Wang G. Antimicrobial peptides: discovery, design and novel therapeutic strategies. England: CAB International; 2010.

[127] Netz DJA, Bastos MCF, Sahl HG (2002). Mode of action of the antimicrobial peptide aureocin A53 from *Staphylococcus aureus*. Appl Environ Microbiol.

[128] Netz DJA, Pohl R, Beck-Sickinger AG, Selmer T, Pierik AJ, Bastos MCF (2002). Biochemical characterisation and genetic analysis of aureocin A53, a new, atypical bacteriocin from *Staphylococcus aureus*. J Mol Biol.

[129] Von Tersch MA, Carlton BC (1983). Bacteriocin from *Bacillus megaterium* ATCC 19213: comparative studies with megacin A-216. J Bacteriol.

[130] Zakharov SD, Cramer WA (2002). Colicin crystal structures: pathways and mechanisms for colicin insertion into membranes. Biochim Biophys Acta.

[131] Michel-Briand Y, Baysse C (2002). The pyocins of *Pseudomonas aeruginosa*. Biochimie.

[132] Bamford CV, Francescutti T, Cameron CE, Jenkinson HF, Dymock D (2010). Characterization of a novel family of fibronectin-binding proteins with M23 peptidase domains from *Treponema denticola*. Mol Oral Microbiol.

[133] Grabowska M, Jagielska E, Czapinska H, Bochtler M, Sabala I (2015). High resolution structure of an M23 peptidase with a substrate analogue. Sci Rep.

[134] Wang H, Fewer DP, Holm L, Rouhiainen L, Sivonen K (2014). Atlas of nonribosomal peptide and polyketide biosynthetic pathways reveals common occurrence of nonmodular enzymes. Proc Natl Acad Sci U S A.

[135] Weissman KJ (2014). The structural biology of biosynthetic megaenzymes. Nat Chem Biol.

[136] Aleti G, Sessitsch A, Brader G (2015). Genome mining: prediction of lipopeptides and polyketides from *Bacillus* and related Firmicutes. Comput Struct Biotechnol J.

[137] Baltz RH (2014). Combinatorial biosynthesis of cyclic lipopeptide antibiotics: a model for synthetic biology to accelerate the evolution of secondary metabolite biosynthetic pathways. ACS Synth Biol.

[138] Meena KR, Kanwar SS. Lipopeptides as the antifungal and antibacterial agents: applications in food safety and therapeutics. Biomed Res Int. 2015. doi: 10.1155/2015/473050. 10.1155/2015/473050 PMC4303012 25632392

[139] Ongena M, Jacques P (2008). *Bacillus* lipopeptides: versatile weapons for plant disease biocontrol. Trends Microbiol.

[140] Cawoy H, Debois D, Franzil L, De Pauw E, Thonart P, Ongena M (2015). Lipopeptides as main ingredients for inhibition of fungal phytopathogens by *Bacillus subtilis/amyloliquefaciens*. Microb Biotechnol.

[141] Raaijmakers JM, De Bruijn I, Nybroe O, Ongena M (2010). Natural functions of lipopeptides from *Bacillus* and *Pseudomonas*: more than surfactants and antibiotics. FEMS Microbiol Rev.

[142] Pathak KV, Keharia H (2013). Identification of surfactins and iturins produced by potent fungal antagonist, *Bacillus subtilis* K1 isolated from aerial roots of banyan (Ficus benghalensis) tree using mass spectrometry. 3 Biotech.

[143] Zhao X, Han Y, Tan XQ, Wang J, Zhou ZJ (2014). Optimization of antifungal lipopeptide production from *Bacillus* sp. BH072 by response surface methodology. J Microbiol.

[144] Malfanova N, Franzil L, Lugtenberg B, Chebotar V, Ongena M (2012). Cyclic lipopeptide profile of the plant-beneficial endophytic bacterium *Bacillus subtilis* HC8. Arch Microbiol.

[145] Abderrahmani A, Tapi A, Nateche F, Chollet M, Leclere V, Wathelet B (2011). Bioinformatics and molecular approaches to detect NRPS genes involved in the biosynthesis of kurstakin from *Bacillus thuringiensis*. Appl Microbiol Biotechnol.

[146] Shoji J, Hinoo H (1975). Chemical characterization of new antibiotics, cerexins A and B. (Studies on antibiotics from the genus *Bacillus*. II). J Antibiot (Tokyo).

[147] Hathout Y, Ho YP, Ryzhov V, Demirev P, Fenselau C (2000). Kurstakins: a new class of lipopeptides isolated from *Bacillus thuringiensis*. J Nat Prod.

[148] Luo C, Liu X, Zhou X, Guo J, Truong J, Wang X (2015). Unusual Biosynthesis and Structure of Locillomycins from *Bacillus subtilis* 916. Appl Environ Microbiol.

[149] Luo C, Liu X, Zhou H, Wang X, Chen Z (2015). Nonribosomal peptide synthase gene clusters for lipopeptide biosynthesis in *Bacillus subtilis* 916 and their phenotypic functions. Appl Environ Microbiol.

[150] Choi SK, Park SY, Kim R, Kim SB, Lee CH, Kim JF (2009). Identification of a polymyxin synthetase gene cluster of *Paenibacillus polymyxa* and heterologous expression of the gene in *Bacillus subtilis*. J Bacteriol.

[151] Huang E, Yousef AE (2014). The lipopeptide antibiotic paenibacterin binds to the bacterial outer membrane and exerts bactericidal activity through cytoplasmic membrane damage. Appl Environ Microbiol.

[152] Ding R, Wu XC, Qian CD, Teng Y, Li O, Zhan ZJ (2011). Isolation and identification of lipopeptide antibiotics from *Paenibacillus elgii* B69 with inhibitory activity against methicillin-resistant *Staphylococcus aureus*. J Microbiol.

[153] Pichard B, Larue JP, Thouvenot D (1995). Gavaserin and saltavalin, new peptide antibiotics produced by *Bacillus polymyxa*. FEMS Microbiol Lett.

[154] Huang Z, Hu Y, Shou L, Song M (2013). Isolation and partial characterization of cyclic lipopeptide antibiotics produced by *Paenibacillus ehimensis* B7. BMC Microbiol.

[155] Qian CD, Wu XC, Teng Y, Zhao WP, Li O, Fang SG (2012). Battacin (Octapeptin B5), a new cyclic lipopeptide antibiotic from *Paenibacillus tianmuensis* active against multidrug-resistant Gram-negative bacteria. Antimicrob Agents Chemother.

[156] Azevedo EC, Rios EM, Fukushima K, Campos-Takaki GM (1993). Bacitracin production by a new strain of *Bacillus subtilis*. Extraction, purification, and characterization. Appl Biochem Biotechnol.

[157] Ducluzeau R, Dubos F, Raibaud P, Abrams GD (1976). Inhibition of *Clostridium perfringens* by an antibiotic substance produced by *Bacillus licheniformis* in the digestive tract of gnotobiotic mice: effect on other bacteria from the digestive tract. Antimicrob Agents Chemother.

[158] Ozcengiz G, Ogulur I (2015). Biochemistry, genetics and regulation of bacilysin biosynthesis and its significance more than an antibiotic. New Biotechnol.

[159] Borisova SA, Circello BT, Zhang JK, van der Donk WA, Metcalf WW (2010). Biosynthesis of rhizocticins, antifungal phosphonate oligopeptides produced by *Bacillus subtilis* ATCC6633. Chem Biol.

[160] Lee JY, Passalacqua KD, Hanna PC, Sherman DH (2011). Regulation of petrobactin and bacillibactin biosynthesis in *Bacillus anthracis* under iron and oxygen variation. Plos One.

[161] Tang Y, Frewert S, Harmrolfs K, Herrmann J, Karmann L, Kazmaier U (2015). Heterologous expression of an orphan NRPS gene cluster from *Paenibacillus larvae* in *Escherichia coli* revealed production of sevadicin. J Biotechnol.

[162] Hansen J, Pschorn W, Ristow H (1982). Functions of the peptide antibiotics tyrocidine and gramicidin. Induction of conformational and structural changes of superhelical DNA. Eur J Biochem.

[163] Kleinkauf H, Gevers W (1969). Nonribosomal polypeptide synthesis: the biosynthesis of a cyclic peptide antibiotic, gramicidin S. Cold Spring Harb Symp Quant Biol.

[164] Kondejewski LH, Farmer SW, Wishart DS, Kay CM, Hancock RE, Hodges RS (1996). Modulation of structure and antibacterial and hemolytic activity by ring size in cyclic gramicidin S analogs. J Biol Chem.

[165] Krätzschmar J, Krause M, Marahiel MA (1989). Gramicidin S biosynthesis operon containing the structural genes *grsA* and *grsB* has an open reading frame encoding a protein homologous to fatty acid thioesterases. J Bacteriol.

[166] Mootz HD, Marahiel MA (1997). The tyrocidine biosynthesis operon of *Bacillus brevis*: complete nucleotide sequence and biochemical characterization of functional internal adenylation domains. J Bacteriol.

[167] Wu XC, Qian CD, Fang HH, Wen YP, Zhou JY, Zhan ZJ (2011). Paenimacrolidin, a novel macrolide antibiotic from *Paenibacillus* sp. F6-B70 active against methicillin-resistant *Staphylococcus aureus*. Microb Biotechnol.

[168] Barsby T, Kelly MT, Andersen RJ (2002). Tupuseleiamides and basiliskamides, new acyldipeptides and antifungal polyketides produced in culture by a *Bacillus laterosporus* isolate obtained from a tropical marine habitat. J Nat Prod.

[169] Patel PS, Huang S, Fisher S, Pirnik D, Aklonis C, Dean L (1995). Bacillaene, a novel inhibitor of procaryotic protein synthesis produced by *Bacillus subtilis*: production, taxonomy, isolation, physico-chemical characterization and biological activity. J Antibiot (Tokyo).

[170] Moldenhauer J, Chen XH, Borriss R, Piel J (2007). Biosynthesis of the antibiotic bacillaene, the product of a giant polyketide synthase complex of the trans-AT family. Angew Chem Int Ed.

[171] Wu L, Wu H, Chen L, Yu X, Borriss R, Gao X (2015). Difficidin and bacilysin from *Bacillus amyloliquefaciens* FZB42 have antibacterial activity against *Xanthomonas oryzae* rice pathogens. Sci Rep.

[172] Chen XH, Vater J, Piel J, Franke P, Scholz R, Schneider K (2006). Structural and functional characterization of three polyketide synthase gene clusters in *Bacillus amyloliquefaciens* FZB 42. J Bacteriol.

[173] Gustafson K, Roman M, Fenical W (1989). The macrolactins, a novel class of antiviral and cytotoxic macrolides from a deep-sea marine bacterium. J Am Chem Soc.

[174] Schneider K, Chen XH, Vater J, Franke P, Nicholson G, Borriss R (2007). Macrolactin is the Polyketide Biosynthesis Product of the pks2 Cluster of *Bacillus amyloliquefaciens* FZB42. J Nat Prod.

[175] Lipomi DJ, Langille NF, Panek JS (2004). Total synthesis of basiliskamides A and B. Org Lett.

[176] Yadav JS, Rao PP, Reddy MS, Prasad AR (2008). Stereoselective synthesis of basiliskamides A and B via Prins cyclisation. Tetrahedron Lett.

[177] Li S, Zhang R, Wang Y, Zhang N, Shao J, Qiu M (2013). Promoter analysis and transcription regulation of fus gene cluster responsible for fusaricidin synthesis of *Paenibacillus polymyxa* SQR-21. Appl Microbiol Biotechnol.

[178] Yu WB, Yin CY, Zhou Y, Ye BC (2012). Prediction of the mechanism of action of fusaricidin on *Bacillus subtilis*. Plos One.

[179] Cochrane SA, Lohans CT, van Belkum MJ, Bels MA, Vederas JC (2015). Studies on tridecaptin B(1), a lipopeptide with activity against multidrug resistant Gram-negative bacteria. Org Biomol Chem.

[180] Sood S, Steinmetz H, Beims H, Mohr KI, Stadler M, Djukic M (2014). Iturin family lipopeptides from the honey bee pathogen *Paenibacillus larvae*. Chembiochem.

[181] Luo Y, Ruan LF, Zhao CM, Wang CX, Peng DH, Sun M (2011). Validation of the intact zwittermicin A biosynthetic gene cluster and discovery of a complementary resistance mechanism in *Bacillus thuringiensis*. Antimicrob Agents Chemother.

[182] Kevany BM, Rasko DA, Thomas MG (2009). Characterization of the complete zwittermicin A biosynthesis gene cluster from *Bacillus cereus*. Appl Environ Microbiol.

[183] Garcia-Gonzalez E, Muller S, Hertlein G, Heid N, Sussmuth RD, Genersch E (2014). Biological effects of paenilamicin, a secondary metabolite antibiotic produced by the honey bee pathogenic bacterium *Paenibacillus larvae*. Microbiologyopen.

[184] Lechner M, Findeiß S, Steiner L, Marz M, Stadler P, Prohaska S (2011). Proteinortho: Detection of (Co-)Orthologs in Large-Scale Analysis. BMC Bioinformatics.

