# Supplementary material for: Identification and classification of known and putative antimicrobial compounds produced by a wide variety of Bacillales species
Source: BMC Genomics. 2016 Nov 7;17:882. doi: 10.1186/s12864-016-3224-y (PMC5100339; doi:10.1186/s12864-016-3224-y)
Supplement: Additional file 2: Table S2. — Characteristics of ribosomally synthesized antimicrobial peptides of Bacillales. (DOCX 201 kb) [file 12864_2016_3224_MOESM2_ESM.docx]

**Table 2** Characteristics of ribosomally synthesized antimicrobial peptides of Bacillales

| Name | Precursor sequence (core peptide in bold) | Gene cluster* | Predicted producer species | Examples |
| --- | --- | --- | --- | --- |
| ***Classification*** | ***Class I small RiPPs*** | ***Subclass 1 Lanthipeptides*** | ***Lanthipeptides class I*** |  |
| Subtilin | MSKFDDFDLDVVKVSKQDSKITPQ**WKSESLCTPGCVTGALQTCFLQTLTCNCKISK** | *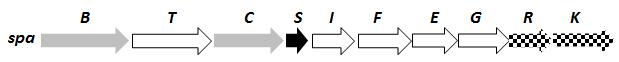* | *Bacillus subtilis*,  *Bacillus* sp. | 26, *Bacillus* sp. YP1 |
| Ericin A | MTNMSKFDDFDLDVVKVSKQDSKITPQ**VLSKSLCTPGCITGPLQTCYLCFPTFAKC** | *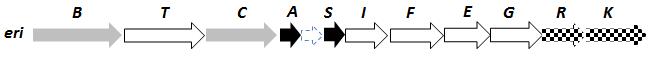* | *B. subtilis* | 46 |
| Ericin S | MSKFDDFDLDVVKVSKQDSKITPQ**WKSESLCTPGCVTGVLQTCFLQTITCNCHISK** | *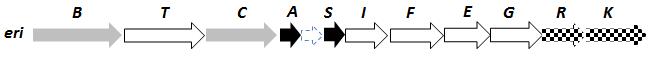* | *B. subtilis* | 46 |
| Entianin | MSKFDDFDLDVVKVSKQDSKITPQ**WKSESLCTPGCVTGLLQTCFLQTITCNCKISK** | 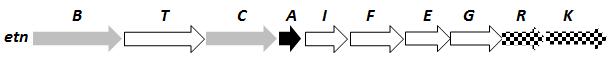 | *B. subtilis* | 45 |
| Subtilomycin | MEKNNIFDLDINKKMESTSEVSAQ**TWATIGKTIVQSVKKCRTFTCGCSLGSCSNCN** | 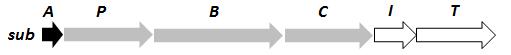 | *B. subtilis* | 42, *B. subtilis* OH 311 |
| Paenibacillin | MKVDQMFDLDLRKSYEASELSPQ**ASIIKTTIKVSKAVCKTLTCICTGSCSNCK** | 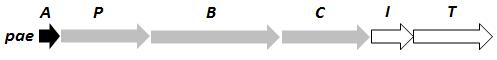 | *Paenibacillus polymyxa* | 43 |
| Thuricin 4A | MNKELFDLDINKKMETPTEMTAQ**TWTTIVKVSKAVCKTGTCICTTSCSNCK** | 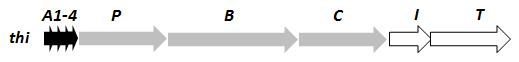 | *Bacillus thuringiensis* | 47 |
| Clausin | MEKAFDLDLEVVHTKAKDVQPD**FTSVSFCTPGCGETGSFNSFCC** | 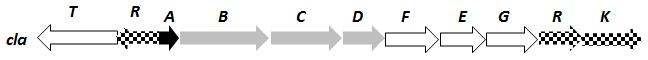 | *Bacillus clausii* | 41, *B. clausii* ENTPro |
| Paenicidin A | MAENLFDLDIQVNKSQGSVEPQ**VLSIVACSSGCGSGKTAASCVETCGNRCFTNVGSLC** | 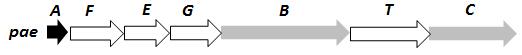 | *P. polymyxa,*  *Paenibacillus terrae* | 44 |
| Paenicidin B | MANNLFDLDVQVNKSQGSVEPQ**VLSIVACSSGCGSGKTAASCVATCGNKCFTNVGSLC** | 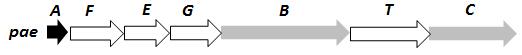 | *P. polymyxa,*  *P. terrae,*  *Paenibacillus* sp. | 44 |
| Geobacillin I | MAKFDDFDLDIVVKKQDDVVQPN**VTSKSLCTGCITGVLMCLTQNSCVSCNSCIRC** | 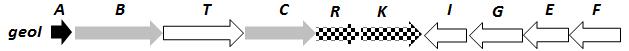 | *Geobacillus thermodenitrificans* | 22, *G. thermodenitrificans* NG80-2 |
| Galidermin/Nisin like lantibiotic | MINEKNLFDLDVQTTASGDVDPQ**ITSISACTPGCGNTGSFNSFCC** | 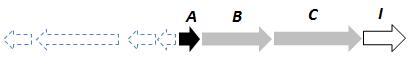 | *Bacillus mycoides,*  *Bacillus cereus* | *B. mycoides* ATCC 6462*, B. mycoides* 2048, *B. cereus* AH1272 |
| Subtilin like lantibiotic | MKNQFDLDLQVAKNEVAPKGVQ**PASGIICTPSCATGTLNCQVSLTFCKTC** | 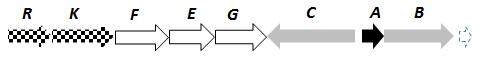 | *P. polymyxa* | *P. polymyxa* SQR 21, *P. polymyxa* CF05, *P. polymyxa* CR1, *P. Polymyxa* E681, *P. polymyxa* M1, *P. polymyxa* SC2 |
| Subtilin like lantibiotic | MKNQFDLDLQVTKSESASKELQ**ADSGIICTPTCLTSILNCYTSISHCGPC** | 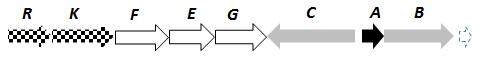 | *P. polymyxa* | *P. polymyxa* M1, *P. polymyxa* SC2 |
| ***Classification*** | ***Class I small RiPPs*** | ***Subclass 1 Lanthipeptides*** | ***Lanthipeptides class II*** |  |
| Mersacidin | MSQEAIIRSWKDPFSRENSTQNPAGNPFSELKEAQMDKLVGAGDMEAA**CTFTLPGGGGVCTLTSECIC** | 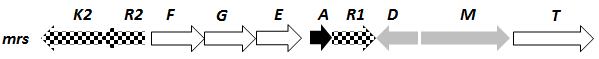 | *Bacillus amyloliquefaciens,*  *Bacillus* sp. | 32, 33, 34, *Bacillus* sp. BH072 |
| Amylolysin | MNEKMYRFAGDLREELEEISLSEFSGGGG**AEQRGISQGNDGKLCTLTWECGLCPTHTCWC** | 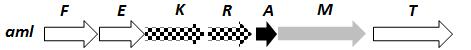 | *B. amyloliquefaciens,*  *Bacillus methylotrophicus* | 50, *B. methylotrophicus* B25, *B. methylotrophicus* NJN-6, *B. amyloliquefaciens* LH15, *B. amyloliquefaciens* LS60 |
| Pseudomycoicidin | MNDKIIQYWNDPAKRSTLSAAELSKMPVNPAGDILAELSDADLDKVVGA**GDCGGTCTWTKDCSICPSWSCWSWSC** | 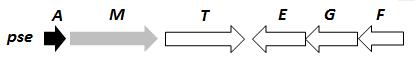 | *Bacillus pseudomycoides* | 51, *B. pseudomycoides* DSM 12442 |
| Cerecidins | MSKGYKFTKEELVEAWKDPQVREKLNDLPKHPSGKALNELSEEELAEIQGA**SDVQPETTPLCVGVIIGLTTSIKICK** | 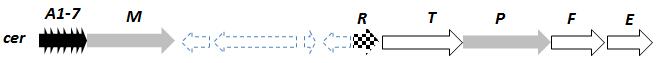 | *B. cereus* | 52 |
| Geobacillin II | MKGGIQMEKQEQTFVSKISEEELKKLAGG**YTEVSPQSTIVCVSLRICNWSLRFPSFKVRCPM** | **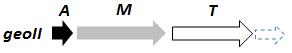** | *G. thermodenitrificans* | 22, *G. thermodenitrificans* NG80-2 |
| Lichenicidin | A1:MSKKEMILSWKNPMYRTESSYHPAGNILKELQEEEQHSIAGG**TITLSTCAILSKPLGNNGYLCTVTKECMPSCN**  A2:MKTMKNSAAREAFKGANHPAGMVSEEELKALVGGNDVNPETTPA**TTSSWTCITAGVTVSASLCPTTKCTSRC** | 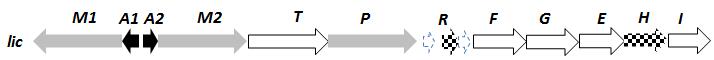 | *Bacillus licheniformis* | 21, *B. licheniformis* WX-02 |
| Haloduracin | A1:MTNLLKEWKMPLERTHNNSNPAGDIFQELEDQDILAGVNGA**CAWYNISCRLGNKGAYCTLTVECMPSCN**  A2:MVNSKDLRNPEFRKAQGLQFVDEVNEKELSSLAGSGDVHAQ**TTWPCATVGVSVALCPTTKCTSQC** | 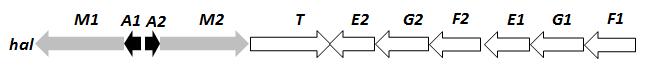 | *Bacillus halodurans* | 19, *B. halodurans* C-125 |
| Cytolysin/ Lichenicidin/ Haloduracin like lantibiotic | A1:MSSKKVVESWKNPVLRSKNEDAPSHPAGEVDSKEIKELFGA**GEGDVTPEGLSSWLGNKGGYCTLTKECMPSCN** A2:MSKNEKLNKLRDQEFDTKELIGSVDENDLKQVAGAGDVNPE**TTPATPTIVAVSLGICPTTKCTSKC** | 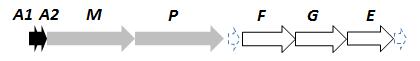 | *B. cereus* | *B. cereus* Q1 |
| ***Classification*** | ***Class I small RiPPs*** | ***Subclass 2 Head to tail cyclized peptides*** |  |  |
| Amylocyclicin | MMNLVKSNKKSFILFGAALAAATLVYALLLTGTELNVAAAHAFSANAE**LASTLGISTAAAKKAIDIIDAASTIASIISLIGIVTGAGAISYAIVATAKTMIKKYGKKYAAAW** | 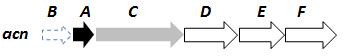 | *B. methylotrophicus* | 59, *B. methylotrophicus* FZB42 |
| Amylocyclicin like circular bacteriocin | MVNSLSNKKRVFLFVVIGLVLATLSSVAYISTLQITIHQTAVLPGNAY**LASTLGISTAAAKKAIDIIDTASTIASIISLIGVVTGAGAISYAVVATAKAMIKKYGKKYAAAW** | 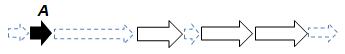 | *Bacillus coagulans* | *B. coagulans* HM08 |
| Uberolysin like circular bacteriocin | M**LEAMGFFGVGKTLATQIVNVVDAVGYAAIAVSTIMAILSAGGLAPTAAAIDFAIIYIKKKIANNLKAQAIVW** | 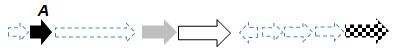 | *Bacillus* sp. | *Bacillus* sp. 1NAL3E |
| Circularin A/ Bacteriocin AS-48 like circular bacteriocin | MSLLA**LVAGTLGVSQSIATTVVSIVLTGSTLISIILGITAILSGGVDAILEIGWSAFVATVKKIVAERGKAAAIAW** | 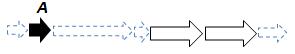 | *Geobacillus stearothermophilus,*  *Geobacillus kaustophilus,*  *Geobacillus* sp. | *G. stearothermophilus* 10*, G. kaustophilus* HTA426*, Geobacillus* sp. C56T3*, Geobacillus* sp. LC300 |
| ***Classification*** | ***Class I small RiPPs*** | ***Subclass 3 Sactipeptides*** |  |  |
| Subtilosin A | MKKAVIVE**NKGCATCSIGAACLVDGPIPDFEIAGATGLFGLWG** | 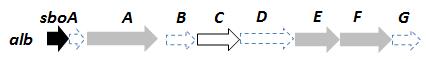 | *B. subtilis,*  *Bacillus atrophaeus,*  *Bacillus smithii,*  *Bacillus* sp. | 67*, B. atrophaeus* subsp. *globigii* BSS*, B. atrophaeus* 1942*, B. atrophaeus* NRS 1221A, *B. atrophaeus* UCMB-5137, *B. smithii* DSM 4216, *Bacillus* sp. JS*, Bacillus* sp. YP1, *Bacillus* sp. BS34A, *Bacillus* sp. LM 4-2 |
| SKF | MKRNQKEWESVSKKGLMKPGGTSIVKAAG**CMGCWASKSIAMTRVCALPHPAMRAI** | 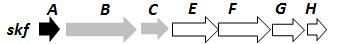 | *B. subtilis,*  *Bacillus pumilus,*  *B. atrophaeus,*  *Bacillus* sp. | 74, *B. pumilus* NJ-M2*, B. pumilus* NJ-V2*, B. atrophaeus* UCMB-5137 |
| Thuricin H(17) | METPVVQPR**DWTCWSCLVCAACSVELLNLVTAATGASTAS** | 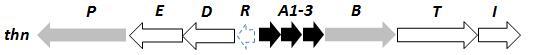 | *B. thuringiensis,*  *B. cereus* | 75, *B. cereus* Rock4-2 |
| Thuricin CD | α:MEVMNNALITKVDEEIGGNAACVIGCIGSCVISEGIGSLVGTAFTLG**GNAACVIGCIGSCVISEGIGSLVGTAFTLG**  β:MEVLNKQNVNIIPESEEVGGWVACVGACGTVCLASGGVGTEFAAASYFL**GWVACVGACGTVCLASGGVGTEFAAASYFL** | 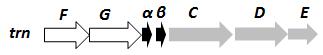 | *B. thuringiensis,*  *B. cereus* | 77, *B. cereus* 95-8201 |
| ***Classification*** | ***Class I small RiPPs*** | ***Subclass 4 LAPs*** |  |  |
| Plantazolicin | MTQIKVPTALIASVHGEGQHLFEPMAA**RCTCTTIISSSSTF** | 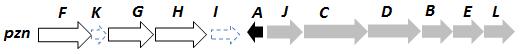 | *B. subtilis,*  *B. amyloliquefaciens,*  *B. methylotrophicus,*  *Bacillus* sp. | 80, *B. subtilis* B1*, B. amyloliquefaciens* CC178*, B. methylotrophicus* JJ-D34*, B. methylotrophicus* FZB42*, B. methylotrophicus* CAU B946*, B. methylotrophicus* UCMB5036*, Bacillus* sp. WP8 |
| ***Classification*** | ***Class I small RiPPs*** | ***Subsclass 5 Thiopeptides*** |  |  |
| Thiocillcin | MSEIKKALNTLEIEDFDAIEMVDVDAMPENEALEIMGA**SCTTCVCTCSCCTT** | 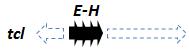 | *B. cereus* | 91 |
| ***Classification*** | ***Class I small RiPPs/*** | ***Subclass 6 Glycocins*** |  |  |
| Sublancin 168 | MEKLFKEVKLEELENQKGS**GLGKAQCAALWLQCASGGTIGCGGGAVACQNYRQFCR** | 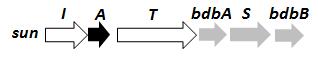 | *B. subtilis,*  *Bacillus* sp. | 92, *Bacillus* sp. BS34A |
| ***Classification*** | ***Class II unmodified bacteriocins*** | ***Subclass1 Pediocin-like peptides*** |  |  |
| Coagulin | MKKIEKLTEKEMANIIGG**KYYGNGVTCGKHSCSVDWGKATTCIINNGAMAWATGGHQGTHKC** | 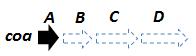 | *B. coagulans* | 109 |
| ***Classification*** | ***Class II unmodified bacteriocins*** | ***Subclass 2 Other unmodified peptides*** |  |  |
| Lichenin | ISLEICXIFHDN | - | *B. licheniformis* | 4 |
| Cerein | MENLQMLTEEELMEIEGG**GWWNSWGKCVAGTIGGAGTGGLGGAAAGSAVPVIGTGIGGAIGGVSGGLTGAATFC** | - | *B. cereus* | 4 |
| Lactobin A family protein | MEGVVFTMELMLEKNGSISFLSEEELKEIDGGRGSWTNAVIGAGTLSPIVASAVRGAQQGVRFGRLGGPWGVVAGAVVGAVVGGYLGYDG | 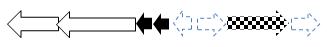 | *Anoxybacillus flavithermus* | *A. flavithermus* WK1 |
| Lactococin A1 family protein | MFQELNHVELQGIDGGSWKSHVVNLVGVVSGFGTTGAVIGGSFGGPLGAAAGGFVGAHYGAVAYAIGVLLDSSNRRK | 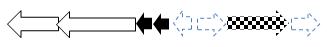 | *A. flavithermus* | *A. flavithermus* WK1 |
| Holin | MDLTSIPIEQFVSNGVFALLFVWLLVDTRKESKQREEKLIQQIEKQNEAQERIVQAIERIEQKIEKLEVSMNG | 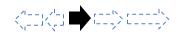 | *Geobacillus* sp. | *Geobacillus* sp. WCH70 |
| BhlA | MEMDISQYLITQGPFAVLFCWLLFYVMKTSKERESKLYDQIDSQNEVLGKFSEKYDVVIEKLDKIESKVQ/MEEQIFNSMIQQGAFAALFVWMLFTTQKKNEQREEQYQKVIEKNQDVITKQAEAFGDLSKDVSEIKQKILGSGDVQ/MEVDVVQNLMTQGPFAVLFCWILFYVLNTTKERENKLNEQIEAQNDVLAKFSEKYDVVIDKLDKIERNLK | 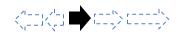 | *B. subtilis,*  *B. thuringiensis,*  *B. pumilus,*  *B. mycoides,*  *B. pseudomycoides,*  *B. amyloliquefaciens* | *B. subtilis* T30*, B. subtilis* subsp. *inaquosorum, B. subtilis* subsp. *spizizenii* NRS 231*, B. thuringiensis* Bt407*, B. thuringiensis* HD1002, *B. thuringiensis* IBL4222*, B. thuringiensis serovar berliner* ATCC 10792*, B. thuringiensis serovar thuringiensis* str. T01001*,* all *B. pumilus* in this study, *B. mycoides* Rock1-4, *B. mycoides* 219298, *B. mycoides* Rock3-17, *B. pseudomycoides* DSM 12442, *B. amyloliquefaciens* DSM 7, *B. amyloliquefaciens* XH7, *B. amyloliquefaciens* MBE1283 |
| LCI | MKFKKVLTGSALSLALLMSAAPAFAASPTASASAENSPISTKADAGIN**AIKLVQSPNGNFAASFVLDGTTWIFKSKYYDSSKGYWVGIYESVDK** | 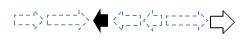 | *B. subtilis,*  *B. amyloliquefaciens,*  *B. methylotrophicus,*  *Bacillus* sp. | 123, *B. amyloliquefaciens* IT-45, *B. amyloliquefaciens* Y2, *B. amyloliquefaciens* CC178, *B. amyloliquefaciens* LFB112, *B. amyloliquefaciens* L-H15, *B. amyloliquefaciens* KHG19, *B. amyloliquefaciens* L-S60, *B. amyloliquefaciens* G341, *B. amyloliquefaciens* MBE1283, all *B. Methylotrophicus* in this study, *Bacillus* sp. BH072 |
| Aureocin A53 | MVAFLKLVAQLGTKAAKWAWDNKSTVINWIKNGATFQWISDKIDSIING | 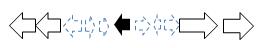 | *B. pumilus* | *B. pumilus* W3, *B. pumilus* GR-8, *B. pumilus* TUAT1 |
| ***Classification*** | ***Class III large antimicrobial proteins*** |  |  |  |
| Colicin | MSLNMYLGQVKAQTESMNAFCNATIQGMEQIIHSINAFALDTVLQGQTYSSAKAYFLQTFRPLAQGIIYLCEELIRQNDAFPRDFQSQVASTDVIEQEILEQIREIDRMIASTEALNQTMPIPGMDAMVNLFTVMRQKLQEKLEHLYEFNYTSSNNYDTALQLAASIATGLAEVQSGKGFSPASGTFSTQGLNMEWTGPIQAITEEKKRKADHLIKDGEMCGRLEETSAIKKAWGDKVDSVVEMFETVKKIWNGTVIGTGKSVEDAIKSMETLSNMNIRNMDIETFINVTYAILHLDETAKNMWHTFSSTVKRDMINGDAESRTQWITYALTQIGIGLIADKGLGRAGLVIKGVKASSGASTLTKGVTLIKEMKHASEILQSFKKDVSYAFSGGTIITKIPQSELNQAYYNFAKTTISSAQKRNSPGTVTSSFNLERSLGTQKKLMYNKGSIGVIPQEIRNKLIGKNFNSFDDFRKEFWKTVADSDYATEFNQRNINLMKEGKAPFAPLSEKYGQHNQYILHHKQPIHQGGDVYNLDNLIIVSPKMHQNVLDRSYHFGKKG | 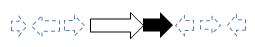 | *B. thuringiensis,*  *B. cereus,*  *Bacillus* sp. | *B. thuringiensis* IBL-200, *B. thuringiensis* serovar morrisoni BGSC 4AA1, *B. thuringiensis* HD-771, *B. thuringiensis serovar sotto str* T04001, *B. thuringiensis* *serovar huazhongensis* BGSC 4BD1, *B. cereus* FORC-005, *B. cereus* m1550, *Bacillus* sp. BH072 |
| Pyocin AP41 | MGQVRVDPDKLESAANSMSRNRESMESIIRELLQVTFELQMSWEGMAYQRFFDEFFSKKRSMDDLVKHLHHTELELKKAAKTFREADEKAFGDFNQMGKLWDAFQRGSGKAAGDTIFDPLKKGWNQTSDFFGNLVDNPMDALEDAKYGLTDFVESSIDDTKEEFRDKYEFMKDMWNNPIGTLKHELDEEVQEMYAIRNVLSDWYVENIKYGDAESITESVAYGATNLAFFGLVTRGASAVGNGARWGKNLSSISKLQLENRLEPAFAYGKIDYKVDTIKPSDTYMFAKTSSAVKRKTPGTVTSSFNLERSLGTQKKLMYNDGAIGIIPQEVREKLVGREFKSFDDFREEFWKTLSDSSYAKEFSPMNIKLMKQGKAPYSPRAEHYGNHNKYILHHKQPIDKGGDVYNLDNLIIVSPKMHQNVLDPAYHFGTKGL | 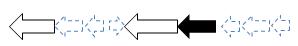 | *B. thuringiensis* | *B. thuringiensis serovar thuringiensis str.* IS5056, *B. thuringiensis serovar chinensis* CT-43 |
| M23 Peptidase | MKKRSILPAAVVCTLSLGGLFGYQSNASAAGDLQQKKSEIESKLSNVQSEMDKKDNQISNIQDKQASLGSQLEAIESKIQSANKKISEQEQNISTTKSQIADLKKNIAEIKKRIEDRNAILEDRARAMQKNGGGSVNYLDVFLEAKSVGDFIDRFSAVKTLVEADRQILEEQKKDEQDLKDKQASVEKKLSDLESMLSDLQSLKDSLKQDESEKSALIKQLDKQKDSLKEEKMSLSEQKSILADQKASIEKAIAFAKKQAEARKEAAAKADAAAKAQTNTNAGSTGSKAGTTSAASSASPSSGSHSGGPLPEVSSGAFTRPASGYISSGFGGRSGGFHPGVDIANSIGTPVVAAADGVVFRAYRSSSYGNCVMITHYINGKLYTTVYAHLSSYSVSTGQHVSKGQQIGAMGNTGESTGPHLHFEIYNGRWTPPPHAGAQNPRNYVNF | 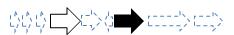 | *B. thuringiensis,*  *B. coagulans,*  *B. halodurans* | *B. thuringiensis* HD-789, *B. thuringiensis* IBL 4222, *B. coagulans* 2-6*, B. halodurans* C-125 |
| Megacins | MKDLNYGLEMIGVKNMWKHSFLGEGVVVAVIDSGAEKEHPAIHSNIIGGFNFTNDDGGEENKYIDYIGHGTHVAGIIAGYDRDLKKIIGVAPLAKLLILKIIDKDGQATIDNACKAIEYALNWRGENGEKVNVMNMSFGTNKDNEHLKSLIKETYSENVIMVASSGNYGDGNALTNELLYPAYYKEVIEVGAVKQNFEIYDYSNSNDEIDFVAPGYKILSSYLNETYVKLSGTSMAAPHVSGAMALLINKFQKEDKEINYKSFYSYLRKDAKKLNYPITLQGHGLIQFKNSKI | 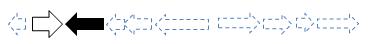 | *Bacillus megaterium* | 129 |

**Structural genes are indicated in black. The genes involved in maturation are indicated in gray. Genes coding for transport and immunity proteins are indicated in white filled solid black box. The white and black grid denotes regulatory genes. The dotted black box denotes gene functions not clearly conserved among the clusters or function unknown. “-” means lack of information. Examples are refered to references of reported peptides and strains of predicted gene clusters in this study.*
