# Supplementary material for: Identification and classification of known and putative antimicrobial compounds produced by a wide variety of Bacillales species
Source: BMC Genomics. 2016 Nov 7;17:882. doi: 10.1186/s12864-016-3224-y (PMC5100339; doi:10.1186/s12864-016-3224-y)
Supplement: Additional file 3: Table S3. — Characteristics of NRPs, PKs and NRPS/PKS hybrid synthesized antimicrobials of Bacillales. (DOCX 854 kb) [file 12864_2016_3224_MOESM3_ESM.docx]

**Table 3** Characteristics of NRPs, PKs and NRPS/PKS hybrid synthesized antimicrobials of Bacillales

| Name | Structure | Gene cluster of NRPS/PKS* | Predicted producer species | Examples |
| --- | --- | --- | --- | --- |
| ***Classification*** | ***NRPs*** | ***Lipopeptides*** |  |  |
| Surfactins  *(e.g. surfactin)* | 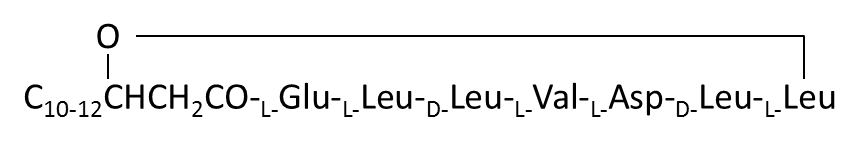 | *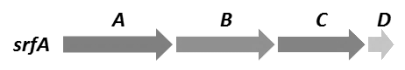* | *Bacillus subtilis*,  *Bacillus amyloliquefaciens,*  *Bacillus methylotrophicus,*  *Bacillus licheniformis,*  *Bacillus paralicheniformis,*  *Bacillus pumilus,*  *Bacillus atrophaeus,*  *Bacillus* sp. | 141, most of the predicted species strains in this study |
| Iturins  *(e.g. iturinA)* | 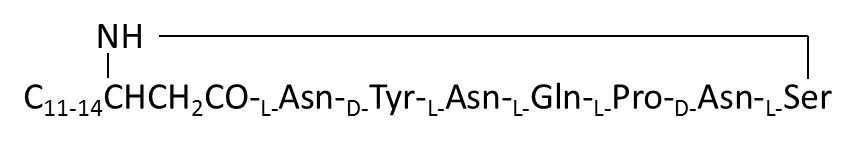 | *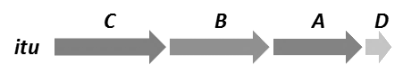* | *B. atrophaeus,*  *Bacillus lehensis,*  *Bacillus* sp.,  *Paenibacillus polymyxa* | 142, all *B. atrophaeus* strains in this study, *B. lehensis* G1, *Bacillus* sp. BH072, *P. polymyxa* Sb3-1, *P. polymyxa* M1, *P. polymyxa* CF05, *P. polymyxa* SC2 |
| Fengycins  *(e.g. fengycin)* | 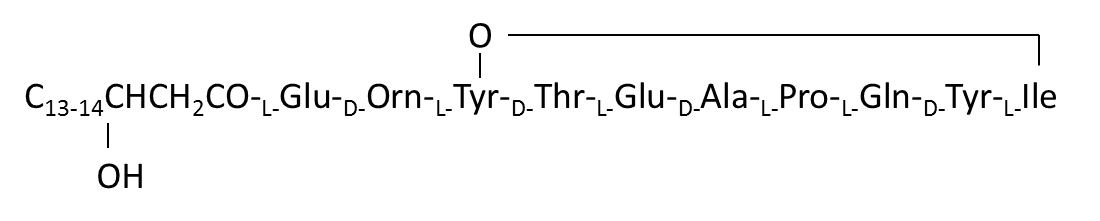 | 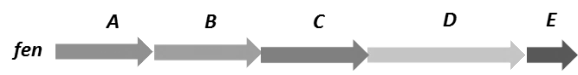 | *B. subtilis*,  *B. amyloliquefaciens,*  *B. methylotrophicus,*  *B. paralicheniformis,*  *Bacillus* sp. | 144, most of the predicted species strains in this study |
| Kustakins  *(e.g. kurstakin)* | 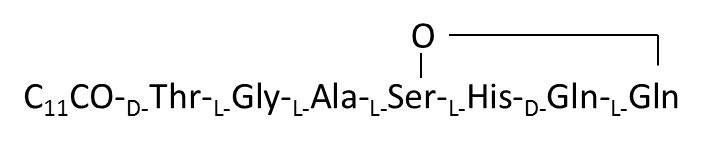 | 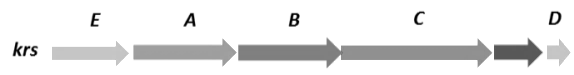 | *Bacillus thuringiensis* | 147 |
| Cerexins  *(e.g. cerexin B)* | 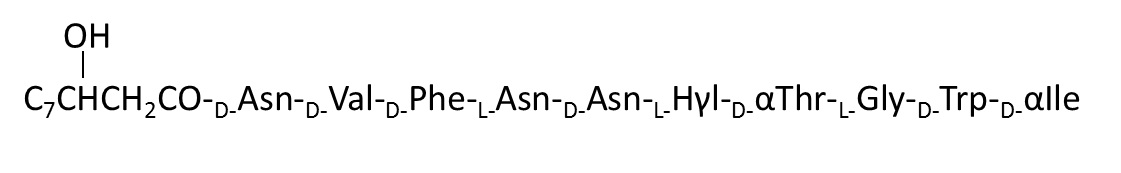 | - | *Bacillus cereus* | 146 |
| Locillomycins  *(e.g. locillomycin)* | 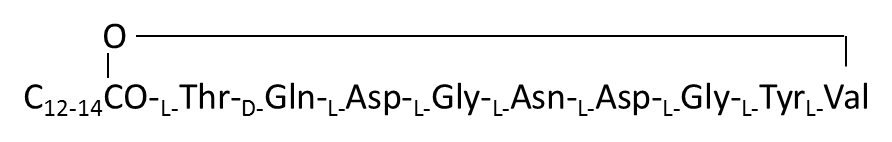 | 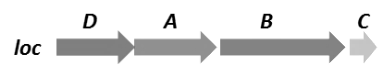 | *B. subtilis*,  *B. amyloliquefaciens,*  *B. methylotrophicus,*  *Paenibacillus mucilaginosus* | 149, *B. subtilis* subsp. *inaquosorum,* *B. amyloliquefaciens* LFB112, *B. methylotrophicus* NJN-6, *P. mucilaginosus* KNP414, *P. mucilaginosus* K02 |
| Polymyxins  *(e.g. polymyxin A)* | 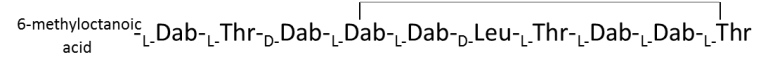 | 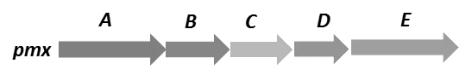 | *P. polymyxa,*  *Paenibacillus peoriae* | 150, *P. polymyxa* SQR-21, *P. polymyxa* Sb3-1, *P. polymyxa* M1, *P. polymyxa* E681, *P. polymyxa* SC2, *P. peoriae* HS311 |
| Paenibacterin | 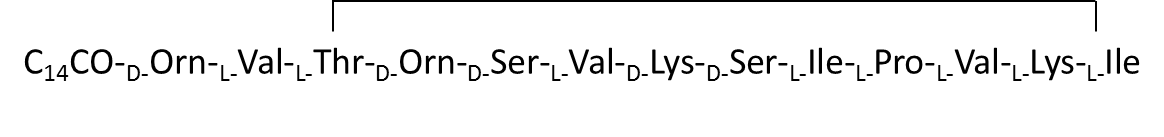 | 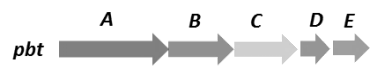 | *Brevibacillus laterosporus,*  *P. mucilaginosus,*  *P. peoriae,*  *Paenibacillus terrae,*  *Paenibacillus* sp. | 151, *B. laterosporus* LMG 15441, *P. mucilaginosus* KNP414, *P. mucilaginosus* 3016, *P. peoriae* HS311, *P. terrae* HPL-003, *Paenibacillus* sp. FSL H7-0357 |
| Fusaricidins  *(e.g. fusaricidin C)* | 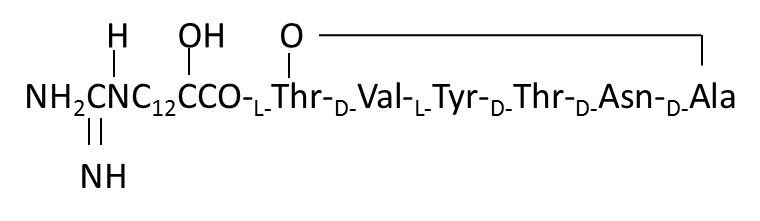 | 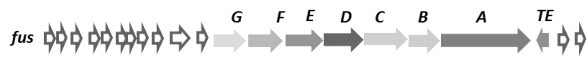 | *P. polymyxa,*  *P. peoriae,*  *Paenibacillus bovis,*  *Paenibacillus* sp. | 136, *P. polymyxa* SQR-21, *P. polymyxa* Sb3-1, *P. polymyxa* M1, *P. polymyxa* E681, *P. polymyxa* SC2, *P. polymyxa* CF05, *P. peoriae* HS311, *P. bovis* BD3526, *Paenibacillus* sp. IHB B 3084 |
| Tridecaptins  *(e.g. tridecaptin A1)* | 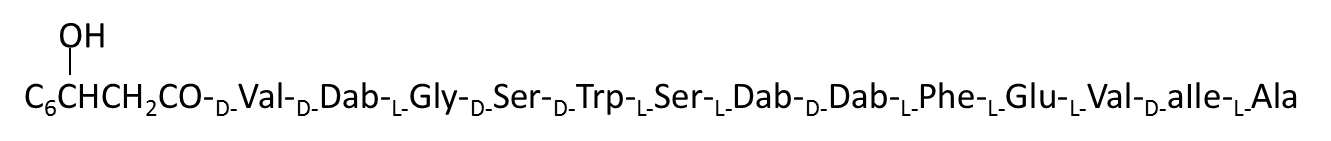 | 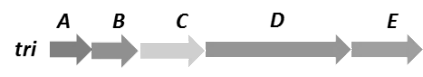 | *P. polymyxa,*  *P. mucilaginosus,*  *P. peoriae,*  *P. terrae,*  *Paenibacillus* sp. | 136, all *P. Polyxyma*, *P. mucilaginosus*, *P. Peoriae* and *P. terrae* strains in this study, *Paenibacillus* sp. IHBB 10380 |
| Polypeptin | 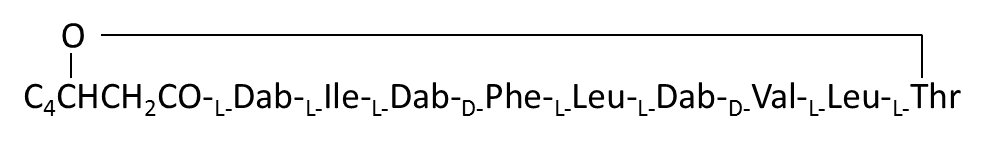 | - | *Paenibacillus* sp. | 154 |
| Pelgipeptins  *(e.g. pelgipeptinC)* | 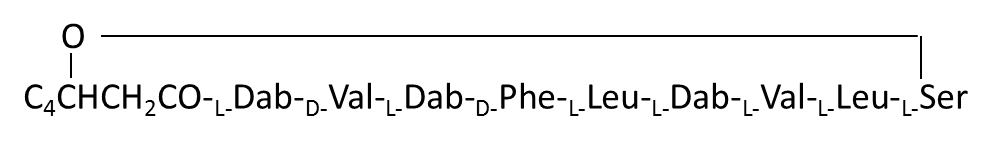 | 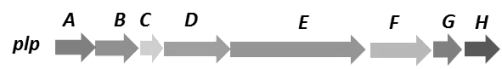 | *Paenibacillus* sp. | 152 |
| Octapeptins  *(e.g. octapeptinD)* | **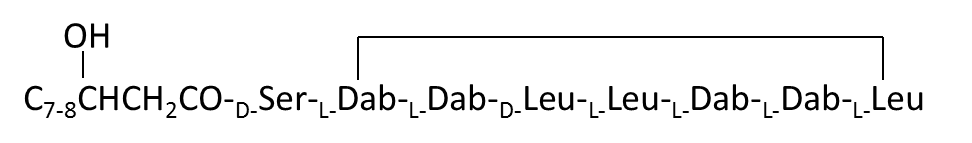** | - | *Paenibacillus* sp. | 155 |
| Gavaserin | - | - | *P. polymyxa* | 153 |
| Saltavalin | - | - | *P. polymyxa* | 153 |
| ***Classification*** | ***NRPs*** | ***Others*** |  |  |
| Bacitracin | 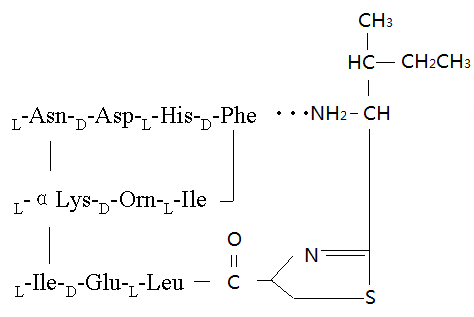 | 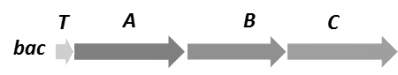 | *B. cereus,*  *B. paralicheniformis,*  *P. bovis,*  *Paenibacillus durus* | 156, 157, *B. cereus* ATCC 10876, *B. cereus* m1550, *B. cereus* Rock3-28, *B. cereus* Rock3-29, *B. cereus* Rock4-18, all *B. paralicheniformis* strains in this study, *P. bovis* BD3526, *P. durus* DSM 1735 |
| Bacilysin | 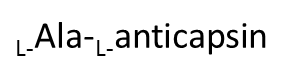 | 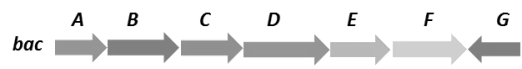 | *B. subtilis*,  *B. amyloliquefaciens,*  *B. methylotrophicus,*  *Bacillus pumilus,*  *Bacillus* sp. | 158, most of the predicted species strains in this study |
| Rhizocticins  *(e.g. rhizocticinA)* | 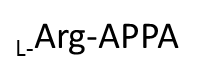 | 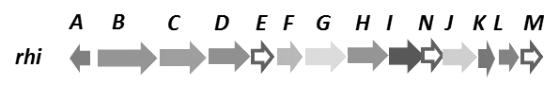 | *B. subtilis*,  *B. atrophaeus,*  *Bacillus* sp. | 159, most of *B. subtilis* and  *B. atrophaeus* strains in this study, *Bacillus* sp. BS34A |
| Petrobactin | 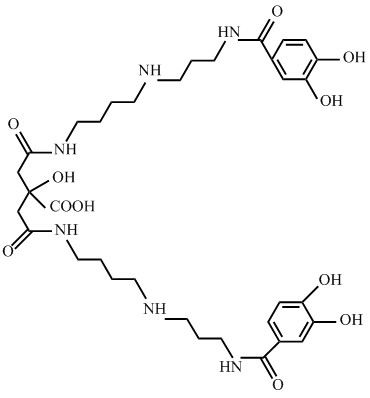 | **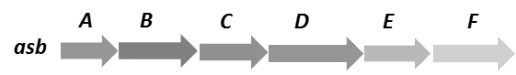** | *B. thurigiensis,*  *B. anthracis,*  *B. cereus,*  *Bacillus mycoides,*  *Bacillus weihenstephanensis,*  *Bacillus bombysepticus,*  *Bacillus toyonensis,*  *B. laterosporus,*  *Brevibacillus brevis,*  *P. mucilaginosus* | 160, most of the predicted species strains in this study |
| Bacillibactin | 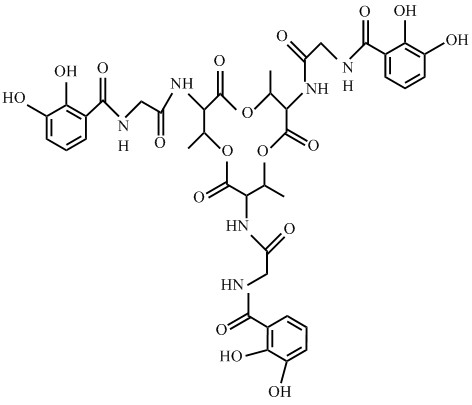 | 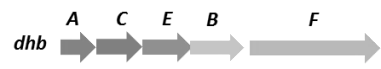 | *B. subtilis,*  *B. thuringiensis,*  *B. anthracis,*  *B. cereus,*  *B. amyloliquefaciens,*  *B. methylotrophicus,*  *B. licheniformis,*  *B. paralicheniformis,*  *B. atrophaeus,*  *B. mycoides,*  *B. weihenstephanensis,*  *Bacillus cytotoxicus,*  *Bacillus endophyticus,*  *Bacillus pseudomcoides,*  *B. bombysepticus,*  *B. toyonensis,*  *Bacillus* sp.,  *Geobacillus* sp.,  *P. larvae,*  *P. bovis,*  *Paenibacillus* sp. | 160, most of the predicted species strains in this study |
| Sevadicin | 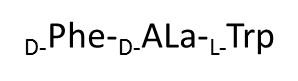 | 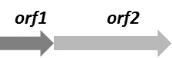 | *P. polymyxa,*  *P. larvae* | 161, *P. polymyxa* SQR-21, *P. larvae* subsp. *larvae* DSM 25430 |
| Gramicidin S | 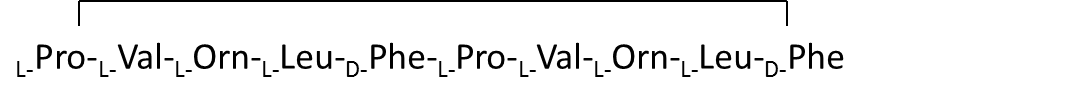 | 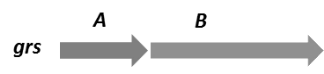 | *B. brevis* | 163, *B. brevis* NBRC 100599 |
| Tyrocidine | 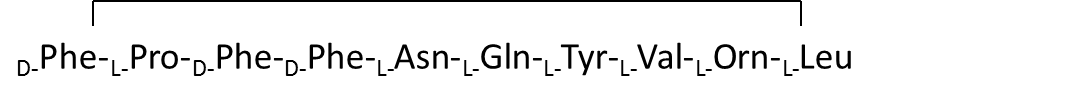 | 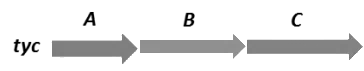 | *B. laterosporus,*  *B. brevis* | 166, *B. brevis* NBRC 100599, *B. laterosporus* LMG 15441 |
| ***Classification*** | ***PKs*** |  |  |  |
| Bacillaene | 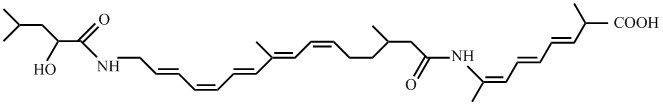 | 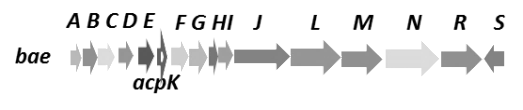 | *B. subtilis,*  *B. amyloliquefaciens,*  *B. methylotrophicus,*  *B. atrophaeus,*  *Bacillus* sp.,  *P. polymyxa*  *P. durus*  *Paenibacillus* sp. | 169, most of the predicted species strains in this study, *Paenibacillus* sp. FSL P4-0081 |
| Difficidin | 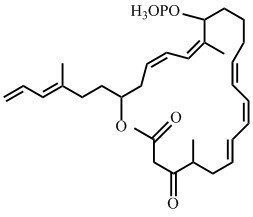 | 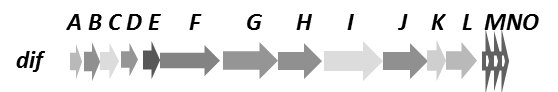 | *B. amyloliquefaciens,*  *B. methylotrophicus,*  *Bacillus* sp.,  *Paenibacillus* sp. | 171, most of the predicted species strains in this study, *Bacillus* sp. BH072, *Bacillus* sp. SDLI1, *Paenibacillus* sp. FSL R7-0273 |
| Macrolactin | 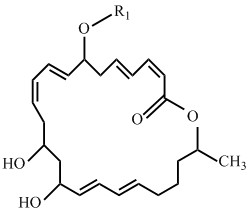 | 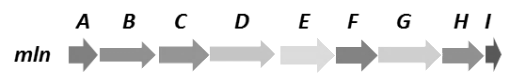 | *B. amyloliquefaciens,*  *B. methylotrophicus,*  *Bacillus* sp. | 173, most of the predicted species strains in this study, *Bacillus* sp. BH072, *Bacillus* sp. SDLI1 |
| Paenimacrolidin | 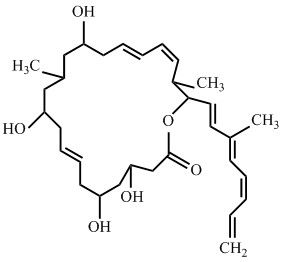 | 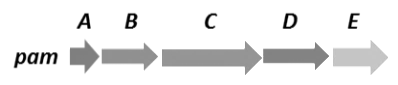 | *Paenibacillus* sp. | 167 |
| Basiliskamides | 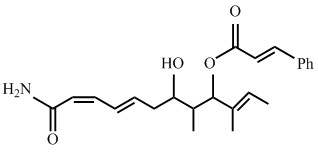 | 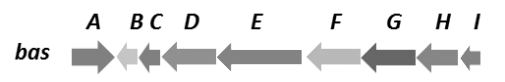 | *B. laterosporus* | 175 |
| ***Classification*** | ***NRPS/PKS hybrid synthesized compounds*** |  |  |  |
| Paenilarvins  *(e.g. paenilarvin A)* | 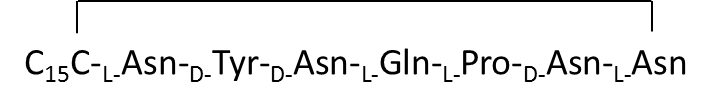 | 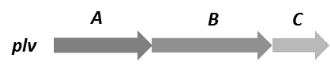 | *P. polymyxa,*  *P. larvae* | 180, *P. polymyxa* SC2, *P. polymyxa* E681, *P. polymyxa* CF05, *P. polymyxa* M1, SQR2-1, *P. larvae* subsp. *larvae* DSM 25430, |
| Zwittermicin A | 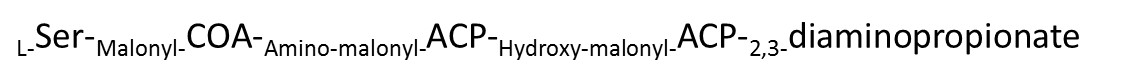 | 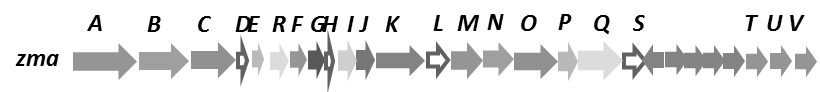 | *B. thurigiensis,*  *B. cereus* | 181, *B. thuringiensis* *serovar thuringiensis str.* IS5056, *B. thuringiensis serovar galleriae* HD-29, B*. thuringiensis serovar kurstaki str.* HD-1, *B. thuringiensis serovar kurstaki str.* YBT-1520, *B. thuringiensis* YC-10, *B. cereus* ATCC 10876, *B. cereus* F65185 |
| Paenilamicins  *(e.g. paenilamicinA1)* | 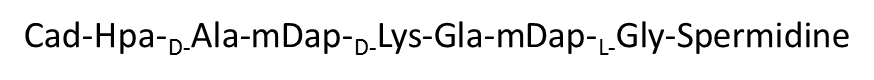 | 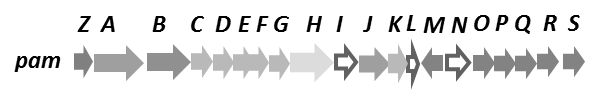 | *B. weihenstephanensis,*  *B. endophyticus,*  *Bacillus gobiensis,*  *P. larvae* | 183, most of the predicted species strains in this study |

**Gene clusters encoding NRPSs or PKSs or NRPS/PKS hybrids are indicated in different arrow boxes. “-” means lack of information. Structures of compounds are drawn by ChemDraw. Examples are refered to references of reported peptides and strains of predicted gene clusters in this study.*
